# Cationic mononuclear ruthenium carboxylates as catalyst prototypes for self-induced hydrogenation of carboxylic acids

**DOI:** 10.1038/ncomms9140

**Published:** 2015-08-28

**Authors:** Masayuki Naruto, Susumu Saito

**Affiliations:** 1 Graduate School of Science, Nagoya University, Chikusa, Nagoya 464-8602, Japan; 2 Institute for Advanced Research, Nagoya University, Chikusa, Nagoya 464-8601, Japan; 3 ACT-C, JST, Chikusa, Nagoya 464-8602, Japan

## Abstract

Carboxylic acids are ubiquitous in bio-renewable and petrochemical sources of carbon. Hydrogenation of carboxylic acids to yield alcohols produces water as the only byproduct, and thus represents a possible next generation, sustainable method for the production of these alternative energy carriers/platform chemicals on a large scale. Reported herein are molecular insights into cationic mononuclear ruthenium carboxylates ([Ru(OCOR)]^+^) as prototypical catalysts for the hydrogenation of carboxylic acids. The substrate-derived coordinated carboxylate was found to function initially as a proton acceptor for the heterolytic cleavage of dihydrogen, and subsequently also as an acceptor for the hydride from [Ru–H]^+^, which was generated in the first step (self-induced catalysis). The hydrogenation proceeded selectively and at high levels of functional group tolerance, a feature that is challenging to achieve with existing heterogeneous/homogeneous catalyst systems. These fundamental insights are expected to significantly benefit the future development of metal carboxylate-catalysed hydrogenation processes of bio-renewable resources.

## Abstract (web summary)

Many structurally diverse carboxylic acids are available from renewable and petrochemical sources. Here, the authors report a method to reduce carboxylic acids to alcohols using hydrogen, with high selectivity, wherein the carboxylates form active and hydrogen-accepting parts of the catalyst system.

## Graphical abstract

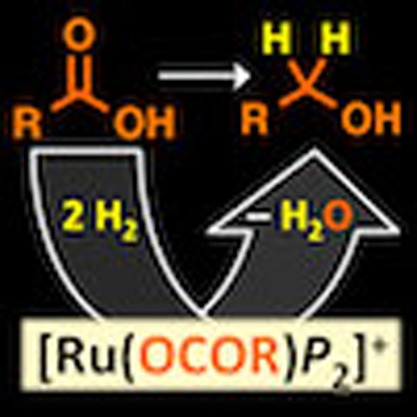

A great variety of carboxylic acids (CAs) is abundantly available from both the petrochemical industry and natural sources. The hydrogenation of CAs is an important research subject with respect to using the resulting alcohols as alternative organic energy (H_2_) carriers, or as platform chemicals^1^. According to a 2004 report from the US Department of Energy, the vast majority of high value-added chemicals from biomass at that time were CAs^2^. While this number has been revised since, due to technological advancements, CAs remain prominent biomass products on this continuously changing list^3^. Alternatively, CAs can be generated synthetically, and an elegant synthesis was recently reported using CO_2_, H_2_ and olefins^4^. Furthermore, formic acid has been produced by hydrogenation of CO_2_ (refs 5, 6, 7, 8), or by the photo-reduction of CO_2_ with H_2_O using solar energy^9^. The availability of carbon-neutral alternatives to fossil fuels presents a critical challenge for the scientific community on the way to a more sustainable society. When CAs are sourced from biomass and/or produced from CO_2_, they represent a potential renewable resource. Finally, recent progress in C–H bond functionalization chemistry has demonstrated the versatility and utility of CAs as effective directing groups in organic synthesis^10^.

The hydrogenation of CAs is an ideal method for the bulk production of alcohols, and may thus benefit the ‘methanol economy', that is, the anthropogenic chemical carbon cycle^11^,^12^, since water is the only byproduct and salt waste is not formed. A hydrogenation method that is widely applicable to a broad variety of CAs and that selectively produces alcohols is therefore highly desirable. Unfortunately, however, simple molecular hydrogenation catalysts that enable such conversions still remain elusive. This is predominantly due to the lack of a rational design strategy for single-active-site catalysts that effectively hydrogenate the thermodynamically stable and kinetically inert COOH group in the absence of undesirable side reactions involving the COOH or other potentially present functional groups. In addition, the catalyst should be able to operate under acidic conditions, given the *ex vi termini* acidity of CAs. This is an important issue, since the catalytic hydrogenation of CA derivatives such as esters^13^,^14^,^15^ and amides^14^,^15^,^16^ proceeds well under basic to neutral conditions, but has seldomly been investigated when using a CA as the acidic reaction medium^15^. Even though conventional approaches to the hydrogenation of CAs with heterogeneous or homogeneous catalysts allow control over a number of reaction parameters, they usually proceed under fair to vigorous conditions (*P*_H2_=2.5–13 MPa and/or *T*=100–500 °C), and they frequently suffer from low alcohol selectivity/productivity^17^,^18^,^19^,^20^,^21^,^22^ as a result of esterification (Ru^0^/Ru^II^)^17^,^18^,^19^, partial reduction to afford aldehydes (Cr_2_O_3_; ref. 20), over reduction (Re^VII^–Os^VIII^; ref. 21), and/or dearomatic hydrogenation (Rh^0^–Mo^0^; ref. 22). Even under milder conditions (typically: *P*_H2_=2–3 MPa, *T*=120–130 °C), decarboxylation (Pt(–Re)/TiO_2_; ref. 23) and esterification (Ir^III^ or Rh^III^; ref. 24) of some CAs represent significant shortcomings. Moreover, studies on functional group tolerance, which may provide useful information for organic synthesis have not yet been reported. In the presence of catalytic Brønsted acids and Ru–triphos complexes^25^,^26^,^27^,^28^,^29^,^30^,^31^,^32^ (triphos=CH_3_C[CH_2_PPh_2_]_3_; Ph=C_6_H_5_), a group of 4-oxo- and 4-hydroxy-CAs, including levulinic acid and succinic acid, undergoes hydrogenation under harsher conditions (*P*_H2_=∼10 MPa, *T*=∼200 °C) to afford alcohols^27^,^28^,^29^,^30^. Very recently, Leitner and co-workers^31^ reported the [Ru(triphos)(TMM)]-catalysed (TMM=trimethylene methane) hydrogenation of acetic acid (AcOH; *P*_H2_=6 MPa, *T*=180 °C), hexanoic acid and benzoic acid (*P*_H2_=5 MPa, *T*=∼220 °C) (ref. 32) in the presence or absence of catalytic amounts of HN(SO_2_CF_3_)_2_ under relatively strenuous conditions. The corresponding alcohols were obtained in moderate to excellent yields (conversion of CAs: 50–99%). However, in many cases, this should be the result of *in situ*-generated esters (lactones) or anhydrides that serve as intermediates, which are able to undergo hydrogenation more effectively^26^,^27^,^28^,^29^,^30^,^31^,^32^,^33^,^34^. For instance, Ru–triphos complexes hydrogenate esters such as methyl benzoate and alkyl formates under milder conditions (for example, *P*_H2_=3–5 MPa, *T*=140 °C) (refs 28, 32). In addition, Goldberg and co-workers^24^ demonstrated experimentally that CAs with shorter aliphatic carbon chains such as AcOH react much more rapidly, which is consistent with previous observations^17^,^18^,^19^. When the size of the CA increases from C_1_ to C_4_, the corresponding carboxylate carbon atom becomes more electron rich and thus less susceptible to nucleophilic attack from transition metal hydrides.

Herein, we would like to report a molecular prototype that benefits from the rational design for a single-active-site Ru catalyst for the hydrogenation of CAs^35^. The CA-derived carboxylate coordinated to the Ru centre was found to function initially as a proton acceptor in the heterolytic cleavage of dihydrogen, and subsequently also as a acceptor for a hydride from [Ru–H]^+^, which was generated in the first step. This catalytic cycle thus represents a CA self-induced CA hydrogenation. This catalyst affords the corresponding alcohols selectively and exhibits a high functional group tolerance. Conversely, the hydrogenation of esters is not promoted, and this catalytic system thus constitutes a benchmark achievement that may be potentially useful for selective organic synthesis and for the development of future catalytic hydrogenation methods for feedstock derived from biomass or CO_2_.

## Results

### Ruthenium carboxylates as catalyst prototypes

**Figure 1:**
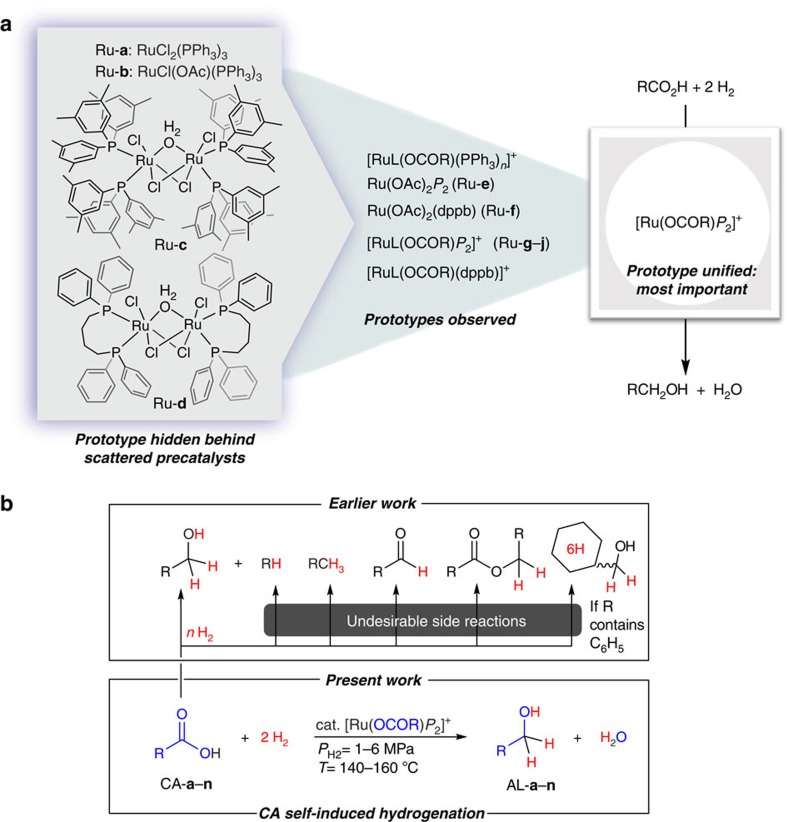
Outline of this study. (**a**) The most favourable R is a saturated aliphatic group. *P*_2_ denotes two monodentate or a bidentate phosphine ligand(s) and the search for optimal *P*_2_ still remains the subject of further optimization. (**b**) CA self-induced CA hydrogenation (present work), in contrast to the shortcomings of earlier CA hydrogenation methods.

Catalyst prototypes (Fig. 1a) for CA self-induced CA hydrogenation, and the characteristics of the present work that differ from earlier studies (Fig. 1b) are shown as the outline of this study.

### Hydrogenation using Ru complex with monodentate phosphine

Since the milestone discovery of the Wilkinson-type ruthenium complex RuCl_2_(PPh_3_)_3_ (Ru-**a**) for the hydrogenation of olefins in the 1960s (refs 36, 37), custom-tailored molecular single-active-site catalysts that hydrogenate CAs effectively have scarcely been investigated systematically^24^,^32^. The hydrogenation of 3-phenylpropionic acid (CA-**a**; Table 1, entry 1) in toluene using a combination of Ru-**a** (2 mol%: [Ru]_0_=6.7 mM), NaBPh_4_ (10 mol%), and high pressure of H_2_ (*P*_H2_=8 MPa, *T*=160 °C, *t*=24 h) resulted in the formation of alcohol AL-**a** and the ester Ph(CH_2_)_2_CO_2_(CH_2_)_3_Ph (ES-**a**) in 58% and 16% yield, respectively (overall conversion of CA-**a**: 92%). In the absence of NaBPh_4_, Ru-**a** was unable to promote the hydrogenation, suggesting that a cationic Ru complex [L_*n*_Ru]^+^ should be of critical importance for a successful hydrogenation. Instead of NaBPh_4_, other similar additives were tested, for example, NaB[3,5-(CF_3_)_2_C_6_H_3_]_4_, NaBF_4_, NaPF_6_, NaOTs, NaOTf and NaNTf_2_, but the results were unsatisfactory (AL-**a**: 0–17%). Encouraged by these results, several commercial derivatives of Ru-**a**, including RuCl_2_(PPh_3_)_4_, RuHCl(CO)(PPh_3_)_3_, RuCl_2_(CO)_2_(PPh_3_)_2_, Ru(*p*-cymene)Cl_2_[P(C_6_H_11_)_3_], Ru(C_5_H_5_)Cl(PPh_3_)_2_, *cis*-RuCl_2_(DMSO)_4_, and Ru(C_5_H_5_)Cl(dppm) (DMSO=dimethylsulfoxide; dppm=1,1-bis(diphenylphosphino)methane) were screened in the presence of NaBPh_4_ (for details, see: Supplementary Table 1). However, the obtained yield of AL-**a** and ES-**a** was consistently below 10%. In contrast, the acetato complex RuCl(OAc)(PPh_3_)_3_ (Ru-**b**; OAc=CH_3_CO_2_^−^) afforded AL-**a** (55%) and ES-**a** (17%) in yields that were similar to those of Ru-**a**. Since a catalytic amount of the Ru-acetato complex Ru-**b** can readily undergo ligand exchange with excess CA-**a** in the reaction mixture, the general formula ‘[Ru(OCOR)]^+^' (R=aliphatic group) is assigned to the critical structure of the catalyst. Although H–H bond scission is known to be promoted by [Ru(OAc)] species^25^,^38^,^39^,^40^, investigations of their potential use have so far been focused more on the hydrogenation of unsaturated carbon–carbon bonds.

To determine the best molar amount of PPh_3_ relative to Ru sufficient to catalyse the hydrogenation of CA-**a**, the precatalyst was switched from Ru-**a** to RuCl_2_(DMSO)_4_ (2 mol%, [Ru]_0_=6.7 mM) and varying molar amounts of the phosphine ligand was used in the presence of 10 mol% NaBPh_4_. The best results, which provided similar hydrogenation rates, were observed for a 2:1 and 3:1 ratio of PPh_3_ and RuCl_2_(DMSO)_4_, while a 1:1 ratio significantly diminished the reaction rate. From the thirteen different monodentate phosphines tested (for details, see: Supplementary Table 2), P(3,5-(CH_3_)_2_(C_6_H_3_))_3_ (P(3,5-xylyl)_3_) afforded the best yield (AL-**a** (ES-**a**): 49% (14%)).

**Figure 2:**
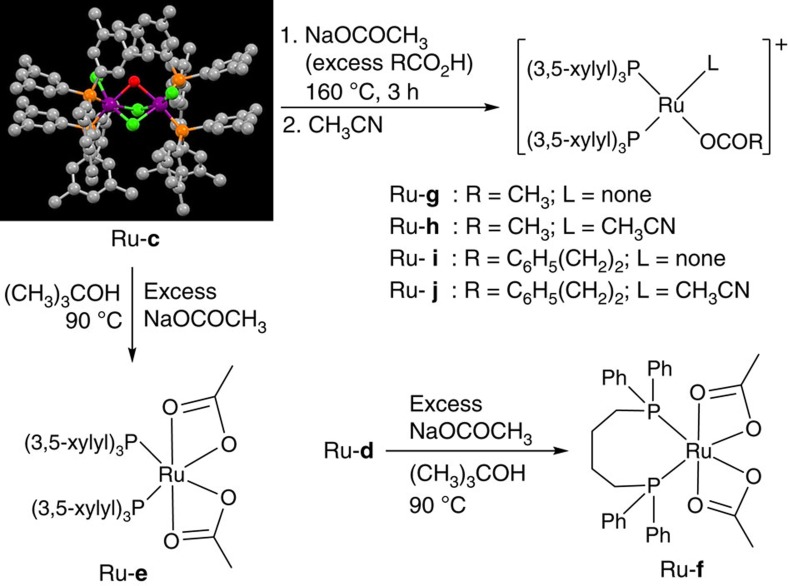
Importance of ‘[Ru(OCOR)]^+^' and its precursors for CA hydrogenation. Single crystal X-ray structure of Ru-**c** (containing ethanol). Colour code: Ru (purple), Cl (green), P (orange), O (red) and C (grey). Calculated exact masses: Ru-**g** (853.2872), Ru-**h** (894.3137), Ru-**i** (943.3341) and Ru-**j** (984.3607).

To gain insight into the structural differences between the [Ru−P(3,5-xylyl)_3_] complex and Ru-**a**, the synthesis of the corresponding Ru complex was attempted by treatment of RuCl_3_·*n*H_2_O with P(3,5-xylyl)_3_ in a molar ratio of 1:6, following the experimental procedure reported for Ru-**a**^36^ (Supplementary Methods). However, the expected Wilkinson-type 16e^–^ complex Ru^II^Cl_2_(P(3,5-xylyl)_3_)_3_ was not obtained. Instead, the binuclear 18e^–^ Ru complex Ru^II^_2_Cl_2_(μ-Cl)_2_(μ-OH_2_)(P(3,5-xylyl)_3_)_4_ (Ru-**c**; see Figs 1a and 2, wherein hydrogen atoms are omitted for clarity) was isolated as a reddish brown precipitate in 83% yield (for the X-ray crystallographic analysis, metric parameters, and an ORTEP structure of Ru-**c**, see: Supplementary Fig. 1). As a solid, Ru-**c** can be easily stored and handled, even under atmospheric conditions. Addition of a third phosphine ligand to the Ru centre, analogous to the formation of Ru-**a** proved to be impossible, presumably due to the sterically congested environment of Ru-**c**. This structural preference is consistent with the observation that a 2:1 ratio between the monodentate phosphine and Ru secured efficient catalysis. When using Ru-**c** (1 mol%) with NaBPh_4_ (10 mol%), hydrogenation of CA-**a** proceeded more effectively, even under a lower hydrogen pressure (*P*_H2_=4 MPa), affording AL-**a** and ES-**a** in 65 and 12% yield, respectively (*T* =160 °C, *t*=24 h; conversion of CA-**a**: 92%). Replacing NaBPh_4_ with NaOAc and Na(acac; acac=acetylacetonate) resulted in comparable effectiveness, furnishing AL-**a** (ES-**a**) in 62% (15%) and 64% (14%) yield, respectively (Supplementary Table 3).

The reaction conditions were further optimized by slightly increasing the load of Ru-**c** to 1.5 mol% so that the hydrogenation was accelerated relative to the simultaneously occurring *in situ* esterification. Furthermore, the more atom-economical NaOAc (10 mol%) was used as the additive for the hydrogenation of various CAs (*P*_H2_=2–4 MPa, *T*=140–160 °C). The results are given in Table 1 (Supplementary Methods for representative methods).

Aliphatic acids CA-**b** and CA-**c** were hydrogenated chemoselectively, exclusively producing alcohols AL-**b** and AL-**c** (entries 2 and 4). This contrasts with the heterogeneous Pt–Re/TiO_2_-catalysed hydrogenation system, in which the decarboxylation of CA-**c** predominates, affording the by one carbon atom diminished alkane^23^. As CA-**d**, was sterically the most demanding CA, we expected it to be kinetically the most inert. Indeed for the hydrogenation of CA-**d**, a slower reaction rate was observed, but AL-**d** was generated cleanly and esterification was not observed (entry 5). CA-**e** (α-phenoxy acid) was one of the most reactive CAs tested, and hydrogenation proceeded smoothly even under relatively mild conditions (*P*_H2_=2 MPa, *T*=140 °C; entry 6). When the hydrogenation of CA-**e** with Ru-**c** (1.5 mol%) and NaOAc (10 mol%) was stopped after 6 h (AL-**e**: 50%), more than 90% of free AcOH (based on added NaOAc) were detected by ^1^H NMR. This result suggests the exclusive formation of a [Ru(OCOCH_2_OPh)]^+^ species, which does not promote the hydrogenation of AcOH, but should be responsible for hydrogenation of CA-**e** (that is, a CA-**e** self-induced CA-**e** hydrogenation). This is in agreement with the previously discussed result, which proposed a [Ru(OCOR)]^+^ complex derived from Ru-**b** as the active catalyst. In contrast, benzoic acid CA-**l** was one of the least reactive substrates (entry 15). And indeed, with the exception of one very recent example^32^, the chemoselective hydrogenation of arenyl (Ar) CAs (ArCO_2_H) using molecular catalysts has so far failed. For instance, both the benzene ring and the carboxyl group of CA-**l** are fully hydrogenated using Rh/Al_2_O_3_–Mo(CO)_6_ as the catalyst system^22^. Relative to aliphatic carboxylates, arenyl carboxylates (^–^OCOAr) such as those derived from CA-**l** should be more strongly coordinated to Ru complexes in a κ^2^-manner^41^,^42^. The nature of the more favourable κ^2^-complexation prevents [Ru(OCOAr)]^+^ from forming a [Ru(η^2^-H_2_)]^+^ species, since the coordination sites of Ru are effectively occupied, rendering the electrophilicity of the Ru centre too low to trap the σ_H–H_ bond. Subsequent formation of a ruthenium hydride, bearing the protonated carboxylate, [RuH(HOCOR)]^+^, is also more favourable with an aliphatic group than with aryl group, that is, the intramolecular deprotonation of η^2^-H_2_ should proceed more readily in the κ^1^-carboxylate structure [Ru(η^2^-H_2_)(κ^1^-OCOR)]^+^ than in the κ^2^-carboxylate, where the carboxylate (R≠Ar) is more basic and thus a less innocent ligand^38^,^39^,^40^,^41^,^42^,^43^,^44^. Indeed, the hydrogenation of CA-**a** in three different initial concentrations ([CA-**a**]_0_=200, 333 and 500 mM) was consistently more sluggish in the presence of a small amount of CA-**l** ([CA-**l**]_0_=67 mM; CA-**l**:Ru-**c**=13:1) than in the absence of CA-**l**, and it also afforded AL-**a** consistently in lower yields. However, irrespective of the presence (*t*=2.5 h) or absence (*t*=1.5 or 2.5 h) of CA-**l**, the reaction rates obtained with the three different initial concentrations [CA-**a**]_0_ in each series were roughly equivalent, providing approximately the same [AL-**a**]_*t*=2.5_ (0.06–0.09 M with CA-**l** versus 0.08–0.11 M without CA-**l**. For the dependence of different [CA-**a**]_0_ with the same [CA-**l**]_0_ on yields of AL-**a** and AL-**l**, see: Supplementary Fig. 2). The hydrogenation rate was observed to be virtually independent of [CA-**a**]_0_, whereby the initially added CA-**l** was recovered effectively (CA-**l**: 78–99%; AL-**l**: 5–10%) in all three runs. These results suggested that the catalytic hydrogenation giving AL-**l** promoted by [Ru(OCOPh)]^+^ via an inner-sphere mechanism was barely involved. The catalysis facilitated by the more reactive [Ru(OCO(CH_2_)_2_Ph)]^+^ via an outer-sphere mechanism (cleavage of H–H bond, followed by an intermolecular hydride transfer from the resulting [RuH(HOCO(CH_2_)_2_Ph)]^+^ to a free CA-**l**) was also negligible. These processes scarcely interfere with the faster catalysis mediated by [Ru(OCO(CH_2_)_2_Ph)]^+^ or [RuH(HOCO(CH_2_)_2_Ph)]^+^ that affords AL-**a** (CA-**a** self-induced CA-**a** hydrogenation in the presence of CA-**l**). To reconfirm that indeed the suspected ‘CA self-induced CA hydrogenation' was observed here, the hydrogenation of CA-**l** was also carried out using three different initial concentrations (*t*=6 h). As in the previous case, a zeroth order rate with respect to [CA-**l**] corroborates a CA-**l** self-induced CA-**l** hydrogenation (Supplementary Fig. 3). These preliminary kinetic experiments indicated that the apparent reaction rate of CA hydrogenation with Ru-**c** and NaOAc at the same parameters for [H_2_] and *T* is independent of [CA]_*t*_ and almost constant, assuming a constant [[Ru(OCOR)]^+^]_*t*_. In other words, the velocity of the rate-determining step should change only on varying the concentration of [Ru(OCOR)]^+^. It is therefore suggested that CA-**a** and CA-**l** should be involved not only as integral ‘carboxylates' of the catalysts for cleaving the H–H bond, but also as ‘protonated carboxylates' that are spontaneously activated and reduced by the resulting [Ru–H]^+^, which is generated in the first step of the CA self-induced CA hydrogenation.

The CO_2_H groups of CA-**h** and CA-**i** were hydrogenated more rapidly than the interior aliphatic and aromatic esters, furnishing AL-**h** and AL-**i** chemoselectively, under preservation of the methyl ester moieties (entries 10 and 12). In addition, when a 1:1 molar mixture of CA-**d** and ethyl stearate (CH_3_(CH_2_)_16_CO_2_CH_2_CH_3_) was hydrogenated using Ru-**c** (1.5 mol%) and NaOAc (10 mol%; *P*_H2_=4 MPa, *T*=160 °C, *t*=24 h), AL-**d** was obtained in 99% yield, while the ester was recovered unchanged. An external ester did not inhibit the catalysis and did not engage with the [RuH(HOCOR)]^+^ species (R=1-adamantyl). The [RuH(HOCOR)]^+^ complex should be accessible only by a CA covalently attached to the Ru centre. In contrast, esters that cannot covalently bind to the Ru complex may have little chance to participate in the catalytic cycle as an integral structure of the catalyst. The applied conditions are most likely not basic enough to effectively promote the homocoupling of alcohols, and therefore the occurrence of a Tishchenko-type reaction giving esters can be excluded (Supplementary Methods). Moreover, it seems that the condensation of the CA with the alcohol to give the corresponding ester is also effectively suppressed in the present system, since the catalyst is unable to hydrogenate esters.

The benzene rings in close proximity to or spatially removed from the CA groups of CA-**a**, **e**, **f**, **i**, **j** and CA-**l–n** were well tolerated (entries 1, 6–8, 12, 13 and 15–18), whereas the amide moiety of CA-**f** (entry 7) and the thiophene moiety of CA-**g** (entry 9) inhibited the catalysis. Despite the fact that the olefin moieties of CA-**i**–**k** were hydrogenated (entries 12–14), the high chemoselectivity and concurrent compatibility of aromatic rings and ester moieties achieved here is virtually unprecedented and rarely achieved with typical, strong, stoichiometric agents such as LiAlH_4_, LiBH_4_, BH_3_ or LiBEt_3_H that reduce not only CAs, but also esters^45^.

### Hydrogenation using Ru complex with bidentate phosphine

To improve the catalytic activity, the combination of a multidentate ligand (2 mol%), RuCl_2_(DMSO)_4_ (2 mol%) and NaBPh_4_ (10 mol%) was examined in detail. From a variety of multidentate ligands screened (Supplementary Table 2), 1,4-(diphenylphosphino)butane (dppb) proved to be one of the best (AL-**a**: 52%, ES-**a**: 14%; *P*_H2_=8 MPa, *T*=160 °C, *t*=24 h). Another elegant approach that differs from ours was reported by Leitner and Klankermayer, who proposed [Ru(triphos)(OCOH)]^+^ and Ru(triphos)(TMM) species as the catalytically active species for the hydrogenation of CO_2_ (*P*_H2_=5 MPa, *T*=140 °C) and CA-**l** (*P*_H2_=50 bar, *T*=220 °C) (ref. 32), giving CH_3_OH (ref. 31) and AL-**l**, respectively. Nevertheless, RuCl_2_(DMSO)_4_/triphos (2 mol% each) was observed to be less effective for CAs with a longer carbon chain (AL-**a**: 22%, ES-**a**: 13%). Moreover, the catalyst systems Ru(acac)_3_/triphos (2 mol% each)^26^,^30^ and [Ru(triphos)(TMM)] (refs 31, 32) (2 mol%) were tested separately (*P*_H2_=8 MPa, *T*=160 °C, *t*=24 h), but the observed reactivity was consistently low (AL-**a**: 22% and 10%, ES-**a**: 10% and 8%, respectively). In contrast, it became clear that a [Ru(dppb)] complex could adopt a structure Ru-**d**^46^, which is similar to Ru-**c**. Hence, Ru-**d** (1 mol%) with Na(acac) (10 mol%) was tested for the hydrogenation of CA-**a** (*P*_H2_=4 MPa, *T*=160 °C, *t*=24 h), which furnished an improved yield of 78 and 10% for AL-**a** and ES-**a**, respectively, under almost quantitative conversion of CA-**a**. Using Ru-**d**, it was also possible to hydrogenate the relatively unreactive substrate CA-**l** under much milder conditions than those reported by Leitner^32^, affording AL-**l** in 93% yield (Table 1, entry 16). The increased catalytic activity of Ru-**d** relative to that of Ru-**c** could also be demonstrated by the following experiment: Ru-**c** and Ru-**d** (1.5 mol% each) were used separately with Na(acac) (10 mol%) for the hydrogenation of CA-**l** (*P*_H2_=4 MPa, *T*=160 °C, *t*=48 h), giving AL-**l** in 26% and 56%, respectively (benzyl benzoate: 2% and 1%, respectively). Substrates CA-**m** and CA-**n**, carrying electron-donating and -withdrawing groups, respectively, were also compatible with these conditions (Table 1, entries 17 and 18). Moreover, the hydrogenation of other substrates that were relatively inert to the Ru-**c**/NaOAc system also proceeded more readily when using Ru-**d** and Na(acac; Table 1, entries 3 and 11). Another advantage of Ru-**d** over Ru-**c** is the structural robustness of the catalyst derived from Ru-**d** under aqueous conditions (Supplementary Table 4).

### Insight into catalytic [Ru(OCOR)*P*_2_]^+^

The prospective resting state of these catalysts, a [Ru(OCOR)*P*_2_]^+^ species, was elucidated by electrospray ionization-mass spectroscopy (ESI–MS) analysis (Fig. 2 and Supplementary Figs 4 and 5). After a toluene solution of a 1:6.7 mixture of Ru-**c** and NaOAc was heated to 160 °C for 3 h, a sample thereof was dissolved in MeCN and examined by ESI–MS, which revealed two primary signals (*m*/*z*=853.2856 and 894.3137) corresponding to Ru-**g** and Ru-**h**, respectively. Presumably, the catalytically innocent species [RuH(CO)[P(3,5-xylyl)_3_]_2_]^+^ (*m*/*z*=823.2752, exact mass: 823.2766) was observed, as its parent compound Ru(OAc)H(CO)[P(3,5-xylyl)_3_]_2_ could be detected by ^31^P{^1^H} NMR in CDCl_3_ (δ 45.8 (s, *P*RuP) and 45.9 (s, PRu*P*) p.p.m. (ref. 47). When a toluene solution of a 1:6.7:33 mixture of Ru-**c**, NaOAc and CA-**a** was heated to 160 °C for 3 h, Ru-**i**, Ru-**j** and Ru-**h** were detected as the primary signals (*m*/*z*=943.3291, 984.3645 and 894.3083, respectively). In both ESI–MS measurements, binuclear structures (involving two Ru centres) could not be detected. The catalytically important structure ‘[Ru(OCOR)*P*_2_]^+^', consistent with Ru-**i** and Ru-**j**, could not be obtained in significant amounts when NaB[3,5-(CF_3_)_2_C_6_H_3_]_4_, NaBF_4_, NaOTf or NaNTf_2_ were used as additives instead of NaOAc or NaBPh_4_ (Supplementary Figs 6 and 7). One of the original Cl^−^ groups of Ru-**c** remained unaffected, or was replaced by CO.

In addition, after a (CH_3_)_3_COH solution of a 1:20 mixture of Ru-**c** and NaOAc was heated to 90 °C for 2 h, Ru-**e** was isolated in 49% yield (Fig. 2; Supplementary Methods). The hydrogenation of CA-**a** with Ru-**e** (3 mol%, [Ru]_0_=10 mM) proceeded effectively (*P*_H2_=4 MPa, *T*=160 °C, *t*=24 h) in the absence of NaOAc, giving AL-**a** (ES-**a**) in 87% (6%) yield, which is comparable to previously obtained results. In the absence of NaOAc (under less basic conditions) and using a small amount of dioxane (10 equiv with respect to CA-**a**), esterification was more effectively suppressed (Table 1, entry 8). Using Ru-**e**, the α,β-unsaturated aliphatic CA tiglic acid (CA-**k**) was hydrogenated at both the olefin and the CO_2_H groups, giving 2-methyl-1-butanol (AL-**k**) in ca. 65% yield (Table 1, entry 14). We would like to use this opportunity to underline the unique reactivity of our system relative to previously described systems: using the comparable Ru-acetato complex Ru(CO)_2_(OCOCH_3_)_2_(PBu_3_)_2_ (Bu=CH_3_(CH_2_)_3_) did not lead to the hydrogenation of the CO_2_H groups of CA-**k** or 2-methylbutanoic acid (*P*_H2_=ca. 13 MPa, *T*=100 °C)^19^. Here the CA self-induced CA hydrogenation via the cationic mononuclear complexes [Ru(OCOR)]^+^ was not observed and the hydrogenation of AcOH was ascribed to ‘unknown catalytic species', affording the ester AcOCH_2_CH_3_. A higher catalytic activity of binuclear Ru-carboxylato complexes relative to that of mononuclear derivatives^18^,^19^ and the importance of intermediary Ru(acyl)(alkoxy) complexes were briefly discussed^18^.

The Ru-diacetato complex Ru-**f** was synthesized from Ru-**d** (Fig. 2)^48^ and was one of the most active Ru catalysts for the hydrogenation of CA-**a** under relatively mild conditions (*P*_H2_=2 MPa; Table 2), even though a slightly decreased yield of AL-**a** was observed under lower hydrogen pressure (*P*_H2_=1 MPa; entry 5). In contrast, hydrogenation with Ru-**e** proceeded comparably, but more selectively using a lower [CA-**a**]_0_ (entry 3 versus 4). The high alcohol selectivity should be ascribed, at least partially, to the low [CA]_0_.

## Discussion

**Figure 3:**
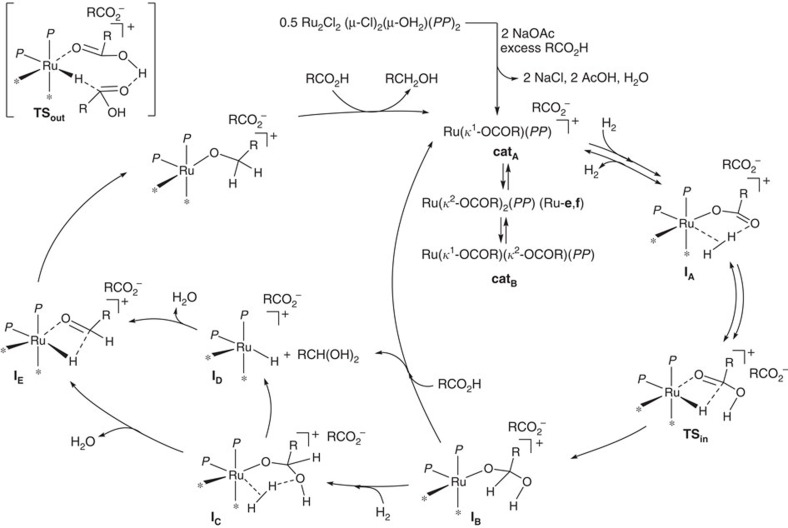
Plausible catalytic cycle involving ‘[Ru(OCOR)*P*_2_]^+^'. *P*_2_ denotes 2P(3,5-xylyl)_3_ or dppb. The asterisk (*) denotes various neutral ligands including solvents such as toluene or dioxane.

On the basis of these observations, we would like to propose a catalytic cycle (Fig. 3).

Important points that corroborate the catalytic cycle can be summarized as: (1) Ru-dicarboxylates such as Ru-**e** and Ru-**f** are stable enough to be isolated; (2) NaBPh_4_ (replacing Cl^−^ in Ru-**c** or Ru-**d** to generate the catalytically active cationic Ru complex) as well as NaOAc effective induced the catalyst; (3) the rate of the hydrogenation was effectively decreased when using Ru-**e** (*P*_H2_=4 MPa, *T*=160 °C, *t*=24 h) in dioxane as a solvent (AL-**a**: 60%; ES-**a**: 2%), which strongly coordinates to the cationic Ru centre; (4) although Ru(CO) complexes were detected by ESI–MS (*vide supra*), the attachment of CO to the Ru centres should be excluded from the catalytic cycle, since such a coordination of CO would be detrimental to the catalytic activity of the CA hydrogenation under mild conditions; and (5) the intermediate ‘[RuH(dppb)]^+^', corresponding to **I**_**D**_ or **I**_**E**_ stabilized by toluene (*m/z* found: 621.1425, calcd: 621.1409) or by CH_3_CN (*m/z* found: 570.1047, calcd: 570.1048) was observed in addition to a [Ru(OCOR)(dppb)]^+^ species analogous to Ru-**i** and Ru-**j**. Those species were generated by treatment of a toluene solution of Ru-**f** with CA-**a** and H_2_ (Ru-**f**:CA-**a** (mol%)=1:33, [CA-**a**]_0_=333 mM, *P*_H2_=4 MPa, *T*=160 °C, *t*=3 h) and were detected in CH_3_CN solution by ESI–MS analysis (for all Ru species observed, see: Supplementary Fig. 8). Another critical intermediate identified was 18e^−^ η^6^-toluene-stabilized **I**_**B**_[Ru(OCH(OH)(CH_2_)_2_Ph)(dppb)(toluene)]^+^ (*m/z* found: 771.2001, calcd: 771.2089). Moreover, [Ru[OCO(CH_2_)_2_Ph](dppb)(CH_3_CN)]^+^ [*m/z* found: 718.1601 (the strongest intensity), calcd: 718.1572] as stabilized **cat**_**A**_ is consistent with the unified ‘prototype [Ru(OCOR)*P*_2_]^+^' in the catalytic cycle involving the CA self-induced CA hydrogenation (**I**_**A**_→**TS**_in_→**I**_**B**_). By taking these facts into account, a cationic ruthenium complex **cat**_**A**_ incorporating one carboxylate is likely the most critical and active species, although catalysis involving the two carboxylate-bearing **cat**_**B**_ can not be fully ruled out. Since the hydrogenation rate using Ru-**e** was affected by the H_2_ pressure (1–4 MPa; Supplementary Fig. 9), the formation of Ru–η^1^-H (**TS**_in_) via Ru–η^2^-H_2_ complex **I**_**A**_ should be the turnover-limiting step in this *P*_H2_ range. A multifunctional (outer sphere) mechanism (**TS**_**out**_; Fig. 3) involving a RCO_2_^–^ moeity^49^ and a RCO_2_H molecule (with a cooperative role for RuH(δ^–^) and Ru[H(δ^+^)OCOR] for hydrogen transfer to another RCO_2_H), as an alternative to a **TS**_**in**_ (inner-sphere mechanism involving direct interaction of the CA with a Ru centre; Fig. 3), might be also possible, although an unambiguous assignment requires a further detailed kinetic and theoretical analysis. Even though free aldehydes were not detected, the release of free hemiacetal from **I**_**C**_ via a four-centered σ-bond metathesis giving **I**_**D**_, or through the hemiacetal–CA exchange of **I**_**B**_ that affords **cat**_**A**_, followed by dehydrative formation of free aldehyde that re-enters the catalytic cycle to give **I**_**E**_, is another possible scenario.

In any case, further refinement of the molecular design of CA hydrogenation catalysts based on the catalytic cycle proposed involves two key factors: (1) the formation of a Ru–η^1^-H species through deprotonation of the Ru–η^2^-H_2_ is facilitated by the participation of a cationic [Ru(OCOR)]^+^ (R=aliphatic group) species. This step may involve a pathway analogous to the concerted metalation–deprotonation mechanism proposed for the cleavage of either H–H (refs 25, 38, 39, 40, 49) or C–H bonds^41^,^42^,^43^,^44^. (2) The CA reformed via the protonation of Ru-carboxylate remains coordinated to the cationic Ru centre of Ru–H species (**I**_**A**_→**TS**_in_). This activated CA in **TS**_in_ is more electrophilic and should be able to facilitate a smooth hydride transfer from the Ru–H species to the carbonyl carbon atom.

In summary, the results obtained from the systematic study of these cationic, mononuclear ruthenium mono-carboxylate catalyst prototypes enabled a rational approach to the design of hydrogenation catalysts for CAs. The results demonstrate that the CAs should act not only as integral carboxylates for the catalysts to cleave the H–H bond, but also as protonated carboxylates that are simultaneously activated and reduced by the resulting [Ru–H]^+^, which is generated in the first step (CA-induced CA hydrogenation). As the availability of bidentate phosphines is virtually infinite, further optimization of the ligand for better catalyst performance under milder conditions is ongoing, together with an extension of the present fundamental research, which has provided this milestone discovery. Identification of ideal metal carboxylates that hydrogenate bio-renewable resources in high oxidation states will significantly benefit the future development of chemical processes directed towards a sustainable society.

## Additional information

**Accession codes**: The X-ray crystallographic coordinate for the structure (Ru-**c**) reported in this Article has been deposited at the Cambridge Crystallographic Data Centre (CCDC), under deposition number CCDC 1024070. The data can be obtained free of charge from The Cambridge Crystallographic Data Centre via www.ccdc.cam.ac.uk/data_request/cif.

**How to cite this article:** Naruto, M. *et al.* Cationic mononuclear ruthenium carboxylates as catalyst prototypes for self-induced hydrogenation of carboxylic acids. *Nat. Commun.* 6:8140 doi: 10.1038/ncomms9140 (2015).

## Supplementary Material

Supplementary Information Supplementary Figures 1-19, Supplementary Tables 1-4, Supplementary Methods and Supplementary References

## Figures and Tables

**Table 1:** Hydrogenation of CAs using catalytic amounts of Ru-**c**, Ru-**d** or Ru-**e**.*

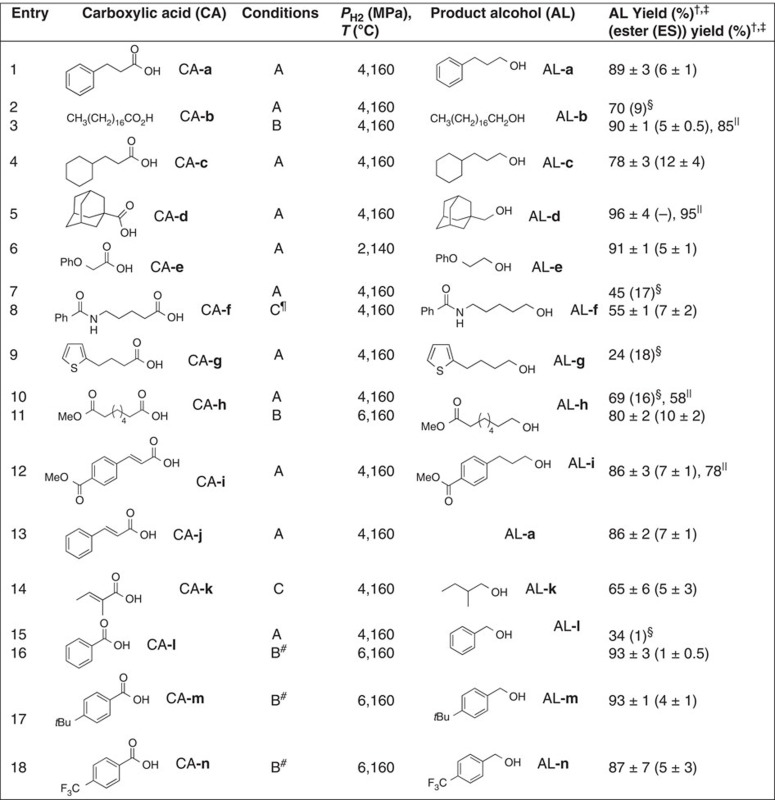

AL, alcohol; CA, carboxylic acid; ES, ester; *T*, temperature.^*^Unless otherwise specified, reactions were carried out over a period of 24 h ([Ru]_0_=10 mM; [CA]_0_=333 mM; in toluene). Conditions A: Ru-**c**:NaOAc:CA=1.5:10:100 mol%; conditions B: Ru-**d**:Na(acac)·(H_2_O)_*n*_:CA=1.5:10:100 mol%; conditions C: 3 mol% Ru-**e** without NaOAc.^†^NMR yields using an internal standard (anisole, mesitylene or 1,1,2,2-tetrachloroethane).^‡^Average of three runs with calculated s.d.^§^Average of two runs.^||^Isolated yield.^¶^In dioxane/toluene (v/v=1:3.5).^#^*t*=48 h.

**Table 2:** Comparison experiments using the Ru(OAc)_2_ *P*_2_ complexes Ru-**c** and Ru-**d** under lower hydrogen pressure (*P*_H2_=1–2 MPa) for the hydrogenation of CA-**a**.*

**Entry**	**Ru complex, mol%**	**Additive, mol%**	**Yield (%)**†‡	
			**AL-a**	**ES-a**
1	Ru-**c**, 1.5	Na(OAc), 10	69	13
2	Ru-**d**, 1.5	Na(acac), 10	69	11
3	Ru-**e**, 3	none	78±3§ (85)||	10±1§ (5)||
4	Ru-**f**, 3	none	76±3§ (80)||	10±1§ (5)||
5¶	Ru-**f**, 3	none	53	11
6	Ru(OAc)_2_[(*R*)-BINAP], 3	none	15	24
7	Ru(OAc)_2_[(*S*)-DMBINAP], 3	none	30	23

AL, alcohol; ES, ester.

^*^Unless otherwise specified, reactions were carried out with CA-**a**:Ru (mol%)=100:3, *P*_H2_=2 MPa, *T*=160 °C for 24 h ([Ru]_0_=10 mM; [CA-**a**]_0_=333 mM; in toluene).

^†^NMR yields are based on the internal standard mesitylene.

^‡^Average of two runs.

^§^Average of three runs with calculated s.d.

^||^([Ru]_0_=5 mM; [CA-**a**]_0_=167 mM). BINAP=2,2'-bis(diphenylphosphanyl)-1,1'-binaphthalene; DMBINAP=2,2'-bis(bis(3,5-dimethylphenyl) phosphanyl)-1,1'-binaphthalene.

^¶^*P*_H2_=1 MPa, *t*=48 h; in dioxane/toluene (v/v=1:3.5).
