# Supplementary material for: Cationic mononuclear ruthenium carboxylates as catalyst prototypes for self-induced hydrogenation of carboxylic acids
Source: Nat Commun. 2015 Aug 28;6:8140. doi: 10.1038/ncomms9140 (PMC4560812; doi:10.1038/ncomms9140)
Supplement: Supplementary Information — Supplementary Figures 1-19, Supplementary Tables 1-4, Supplementary Methods and Supplementary References [file ncomms9140-s1.pdf]

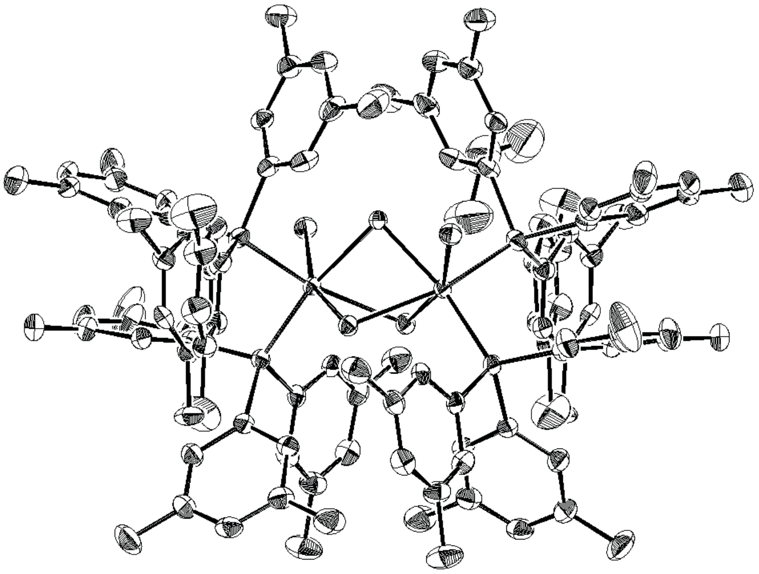

Supplementary Figure 1 | ORTEP drawing of Ru-**c** (50% probability ellipsoids). Hydrogen atoms were omitted for clarity. See S46 for crystal data.

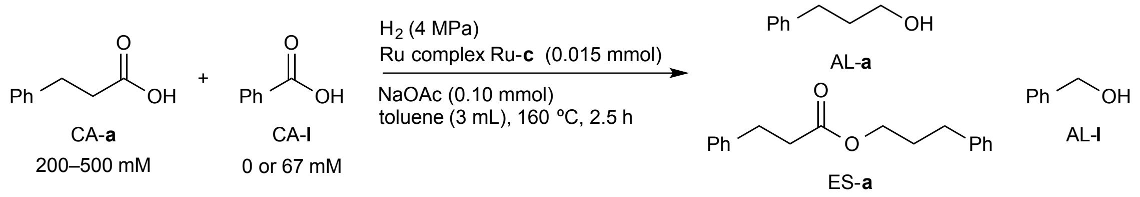

Enrty	$[\text{CA-}\mathbf{a}]_0$ (mM)	$[\text{CA-}\mathbf{l}]_0$ = 0 mM: $[\text{AL-}\mathbf{a} + \text{ES-}\mathbf{a}]_{t = 2.5\ h}$[a, b] (M)	$[\text{CA-}\mathbf{l}]_0$ = 67 mM: $[\text{AL-}\mathbf{a} + \text{ES-}\mathbf{a}]_{t = 2.5\ h}$[a, b] (M)	$[\text{CA-}\mathbf{l}]_0$ = 67 mM: $[\text{AL-}\mathbf{l}]_{t = 2.5\ h}$[a] (M)	$[\text{CA-}\mathbf{l}]_0$ = 67 mM: [Remained $\text{CA-}\mathbf{l}]_{t = 2.5\ h}$[a] (M)
1	200	0.084 ± 0.01	0.058 ± 0.002	0.0067 ± 0	0.052 ± 0.002
2	333	0.098 ± 0.02	0.066 ± 0.004	0.0033 ± 0	0.062 ± 0.002
3	500	0.11 ± 0.01	0.087 ± 0.003	0.0033 ± 0	0.066 ± 0.002

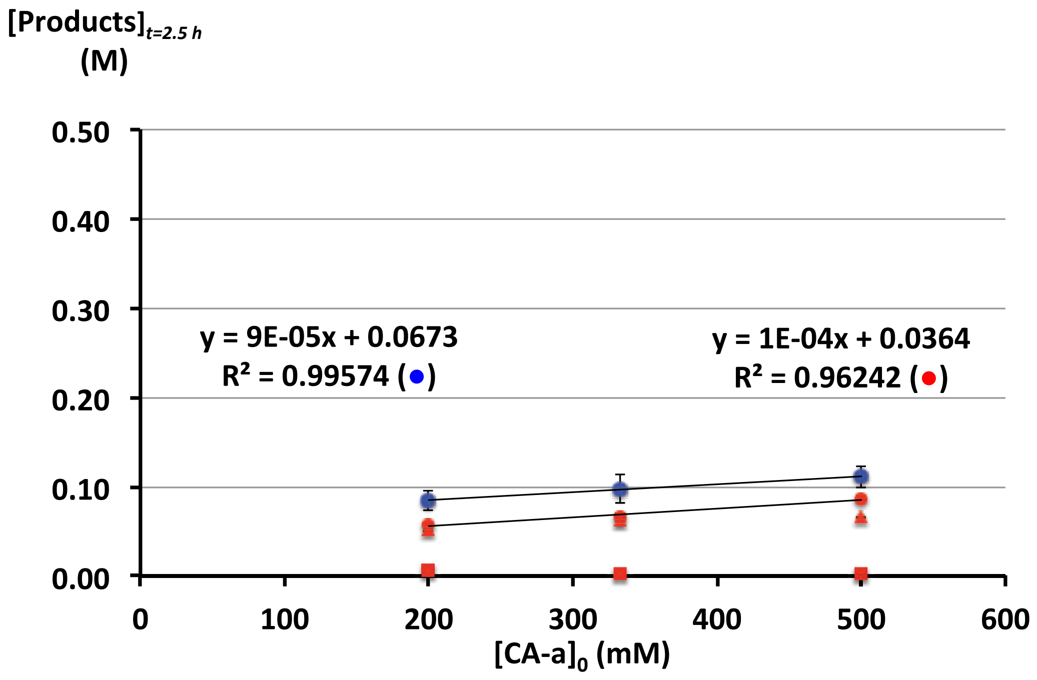

Supplementary Figure 2 | Substrate (CA-**a**) concentration dependency on yields of AL-**a** and ES-**a** using Ru complex Ru-**c**.

Unless otherwise specified, the reactions were carried out with Ru-**c**:NaOAc:CA-**a** (mol %) = 1.5:10:60 or 1.5:10:100 or 1.5:10:150, P_{H2} = 4 MPa, T = 160 °C, and t = 2.5 h. Blue circle, red circle, red square and red triangle represent the concentration of AL-**a** + ES-**a** in the absence of CA-**l** (●), AL-**a** + ES-**a** in the presence of CA-**l** (●), AL-**l** (■) and remained CA-**l** (▲), respectively.

[a] The concentration of products were determined by ^{1}H NMR analysis based on internal standard (mesitylene). The concentrations of products are average of three trials with calculated standard deviation.

[b] The concentrations of ester ES-**a** were less than 0.01 M in all entries.

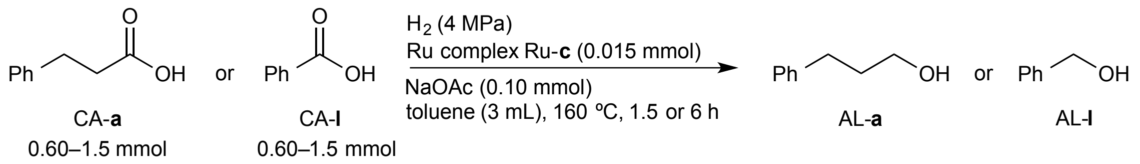

Enrty	CA-**a** (mmol)	[CA-**a**]$_0$ (mM)	AL-**a**[a] (mmol)	[AL-**a**]$_{t=1.5\ h}$ (M)	Enrty	CA-**l** (mmol)	[CA-**l**]$_0$ (mM)	AL-**l**[a] (mmol)	[AL-**l**]$_{t=6\ h}$ (M)
1	0.60	200	0.11	0.037	4	0.60	200	0.12	0.040
2	1.0	333	0.13	0.043	5	1.0	333	0.14	0.047
3	1.5	500	0.15	0.050	6	1.5	500	0.14	0.047

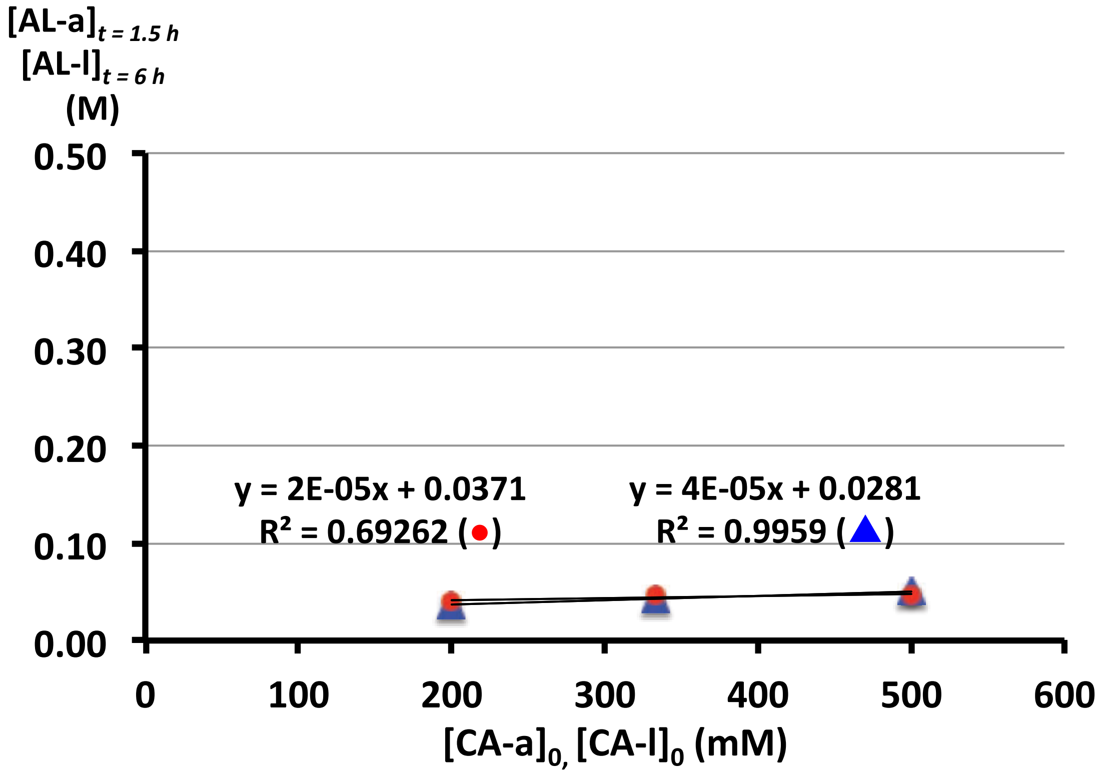

Supplementary Figure 3 | Substrate (CA-**a** or CA-**l**) concentration dependency on yields of AL-**a** and AL-**l** using Ru complex Ru-**c**.

Unless otherwise specified, the reactions were carried out with Ru-**c**:NaOAc:CA (mol %) = 1.5:10:60 or 1.5:10:100 or 1.5:10:150, P_{H2} = 4 MPa, T = 160 °C, and t = 1.5 h for CA-**a** and t = 6 h for CA-**l**. Blue triangle and red circle represent the concentration of AL-**a** (▲) at 1.5 h and AL-**l** (●) at 6 h, respectively. Negligible amount of side products (ester ES-**a** and ES-**l)** were detected in ^{1}H NMR.

[a] The amount of AL-**a** and AL-**l** were determined by ^{1}H NMR analysis based on internal standard (mesitylene).

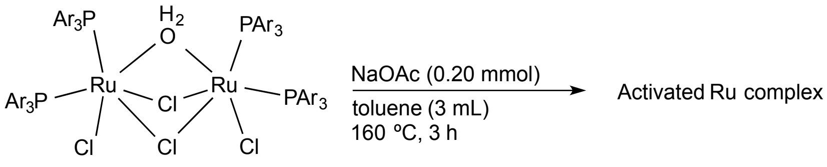

(Ar = 3,5-xylyl)
Ru complex Ru-**c**
0.030 mmol

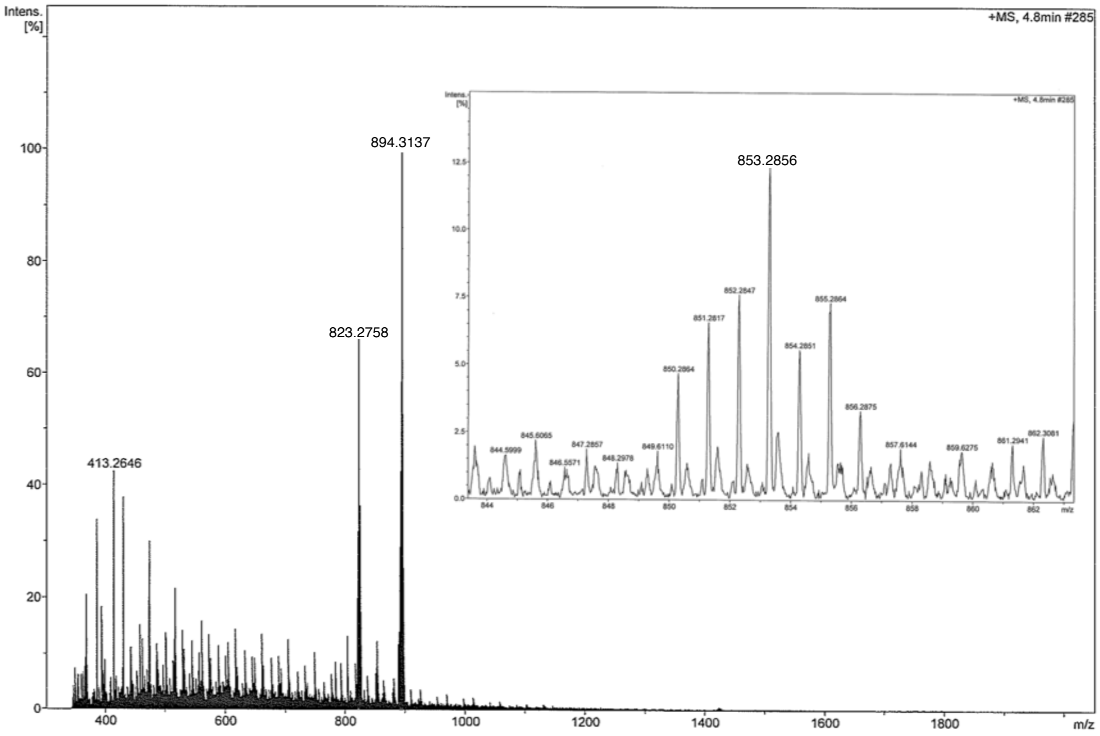

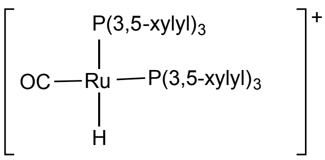

Exact Mass: 823.2766

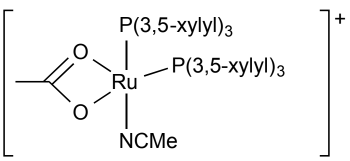

Ru-**h**
Exact Mass: 894.3137

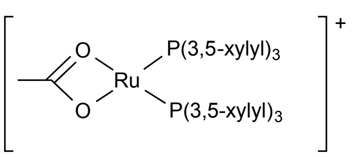

Ru-**g**
Exact Mass: 853.2872

Supplementary Figure 4 | ESI-MS spectrum of a mixture of Ru species ($[\mathrm{Ru}]_0$ = 20 mM, $[\mathrm{NaOAc}]_0$ = 67 mM, T = 160 °C, and t = 3 h).

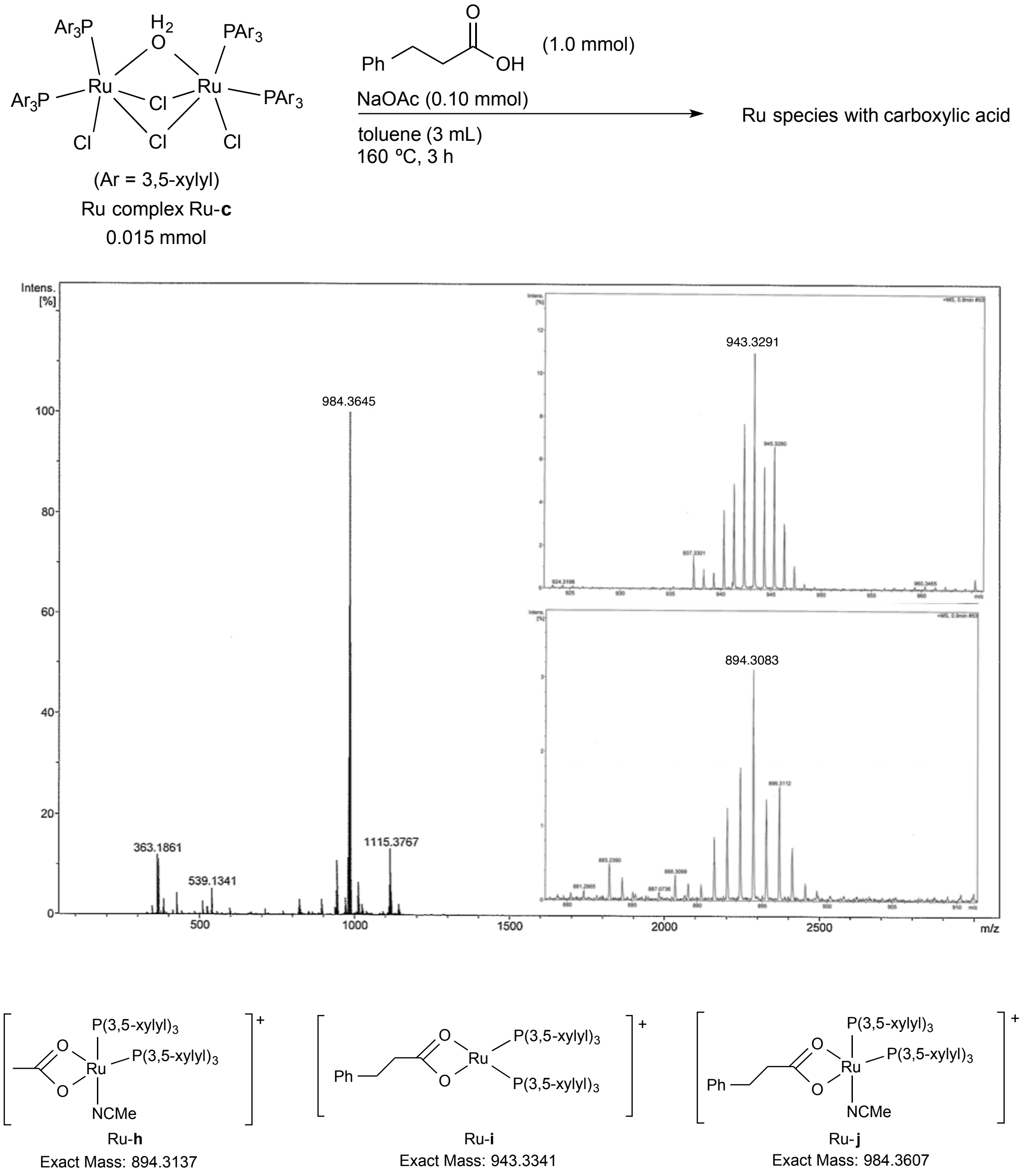

Supplementary Figure 5 | ESI-MS spectrum of a mixture of Ru species ($[Ru]_0$ = 10 mM, $[NaOAc]_0$ = 33 mM, [CA-**a**]$_0$ = 333 mM, T = 160 °C, and t = 3 h).

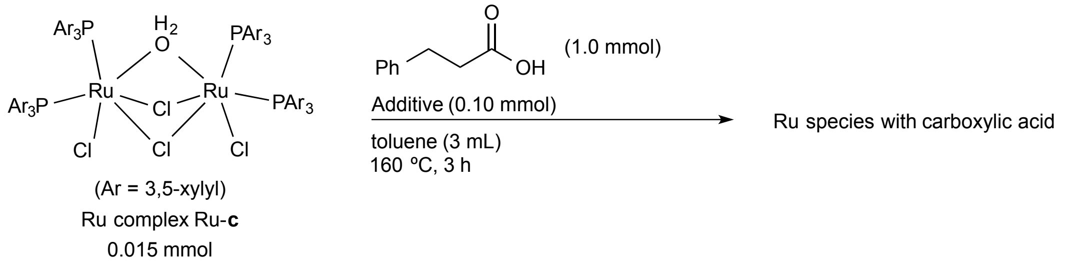

Additive: $NaBPh_4$ (effective) — $P = P(3,5\text{-xylyl})_3$, $R = (CH_2)_2Ph$

Observed *m/z* (Intensity)	823.2727 (40%)	943.3266 (20%)	971.3292 (90%)	984.3602 (100%)	1012.3542 (70%)
Candidates (Exact mass)	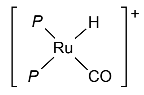 (823.2766)	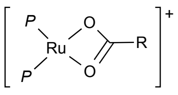 (943.3341)	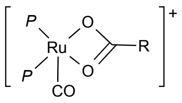 (971.3290)	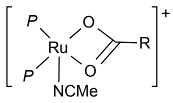 (984.3607)	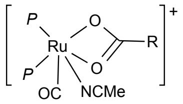 (1012.3556)

Additive: $NaB[3,5\text{-}(CF_3)_2C_6H_3]_4$ (not effective)

Observed *m/z* (Intensity)	889.3461 (100%)	971.3279 (40%)
Candidates (Exact mass)	Not identified	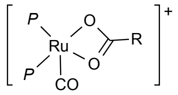 (971.3290)

Additive: $NaBF_4$ (not effective)

Observed *m/z* (Intensity)	829.2438 (100%)	857.2399 (100%)	870.2630 (30%)	971.3229 (15%)	984.3581 (30%)
Candidates (Exact mass)	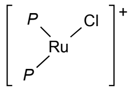 (829.2427)	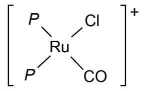 (857.2376)	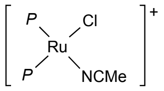 (870.2693)	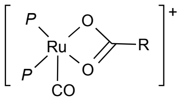 (971.3290)	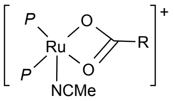 (984.3607)

Additive: NaOTf (not effective)

Observed *m/z* (Intensity)	829.2448 (100%)	857.2397 (90%)	870.2664 (25%)	887.3439 (40%)	921.3037 (40%)	984.3562 (10%)
Candidates (Exact mass)	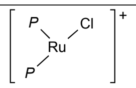 (829.2427)	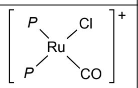 (857.2376)	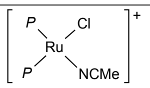 (870.2693)	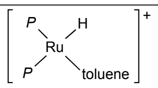 (887.3443)	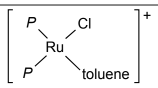 (921.3053)	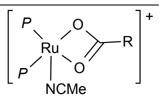 (984.3607)

Additive: $NaNTf_2$ (not effective)

Observed *m/z* (Intensity)	829.2470 (10%)	857.2435 (25%)	887.3523 (50%)	921.3166 (100%)
Candidates (Exact mass)	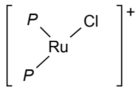 (829.2427)	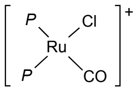 (857.2376)	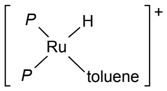 (887.3443)	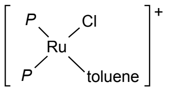 (921.3053)

Supplementary Figure 6 | ESI-MS spectra of a mixture of Ru species ($[Ru]_0$ = 10 mM, $[Additive]_0$ = 33 mM, [CA-**a**]$_0$ = 333 mM, T = 160 °C, and t = 3 h).

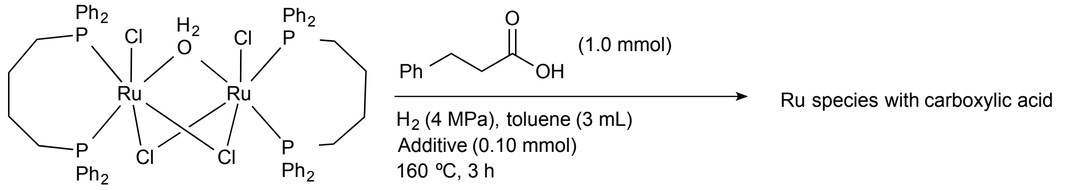

Ru complex Ru-**d**

0.015 mmol

Additive: Na(acac)•xH_2O (effective) *P–P* = dppb, R = $(CH_2)_2Ph$

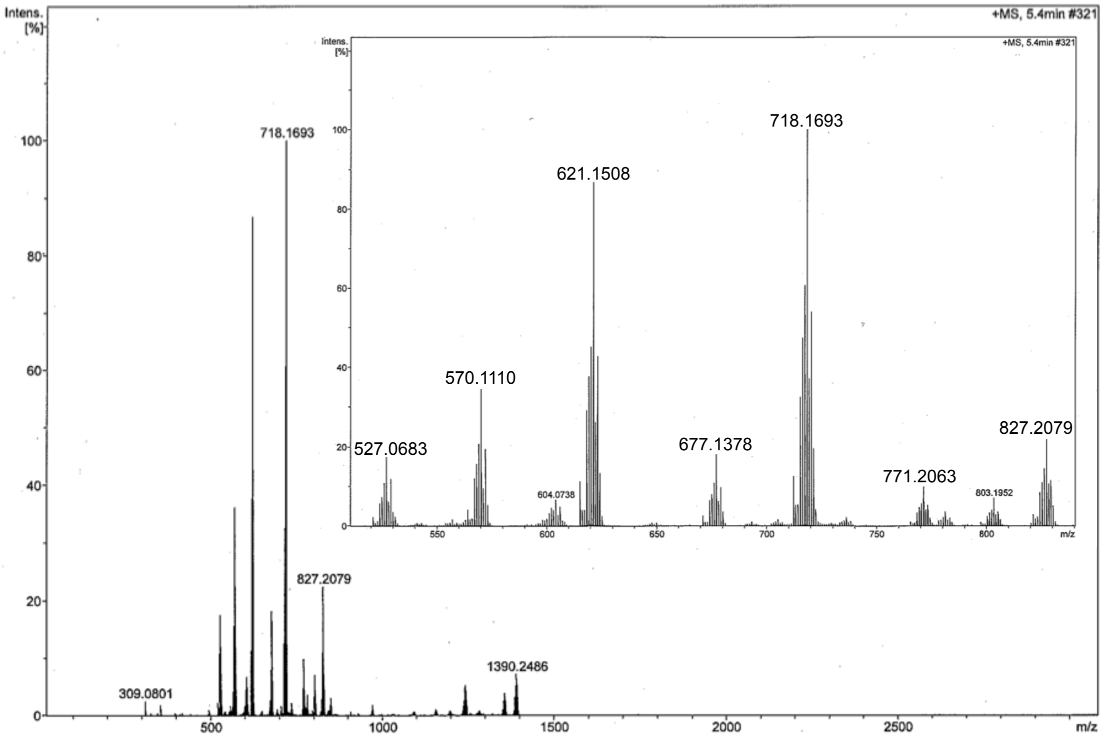

Observed *m/z* (Intensity)	527.0683 (20%)	570.1110 (40%)	621.1508 (90%)	677.1378 (20%)	718.1693 (100%)	827.2079 (30%)
Candidates (Exact mass)	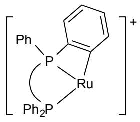 (527.0626)	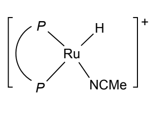 (570.1048)	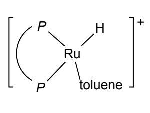 (621.1409)	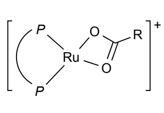 (677.1307)	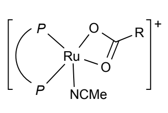 (718.1572)	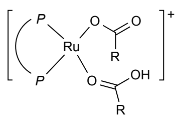 (827.1988)

Additive: $NaB[3,5-(CF_3)_2C_6H_3]_4$ (not effective)

Observed *m/z* (Intensity)	478.0718 (60%)	621.1376 (100%)
Candidates (Exact mass)	Not identified	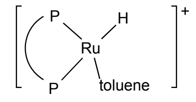 (621.1409)

Additive: $NaNTf_2$ (not effective)

Observed *m/z* (Intensity)	621.1401 (100%)	655.0996 (90%)
Candidates (Exact mass)	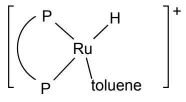 (621.1409)	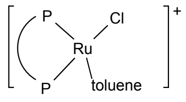 (655.1019)

Supplementary Figure 7 | ESI-MS spectra of a mixture of Ru species ($[Ru]_0$ = 10 mM, $[Additive]_0$ = 33 mM, [CA-**a**]$_0$ = 333 mM, T = 160 °C, and t = 3 h).

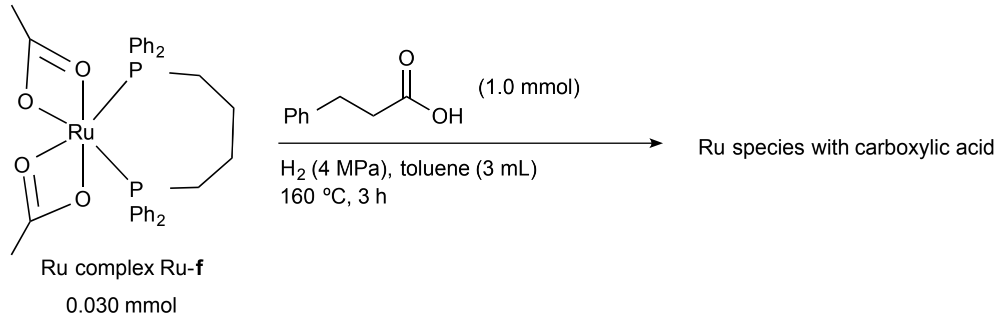

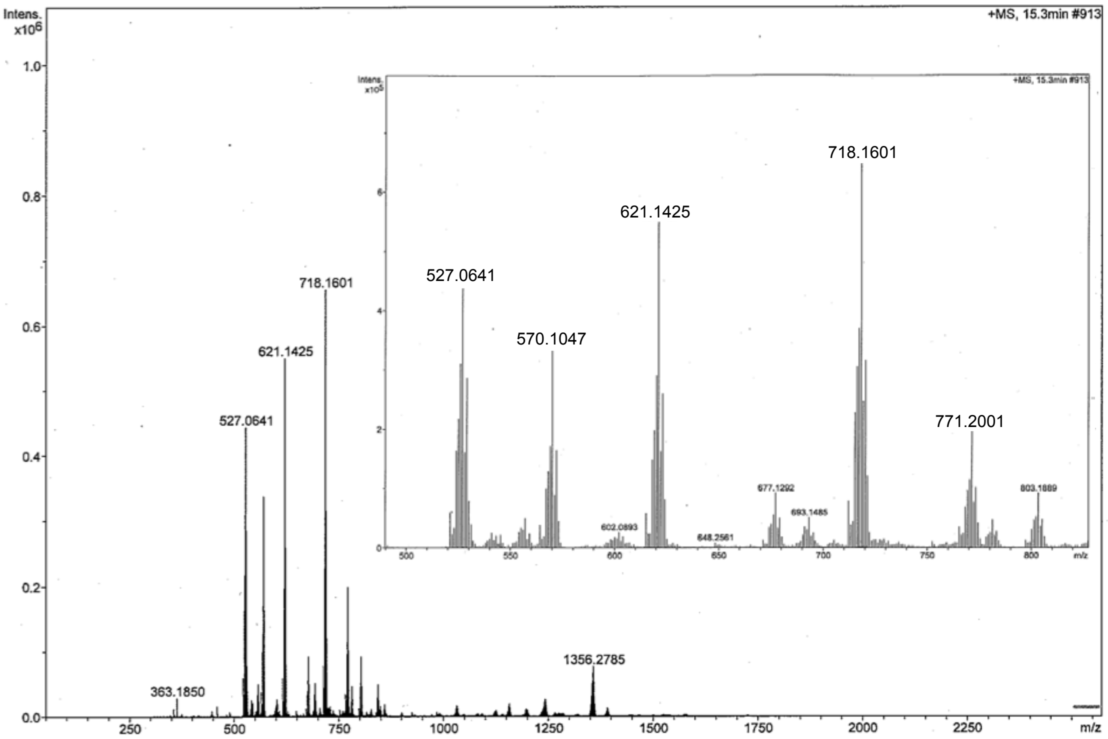

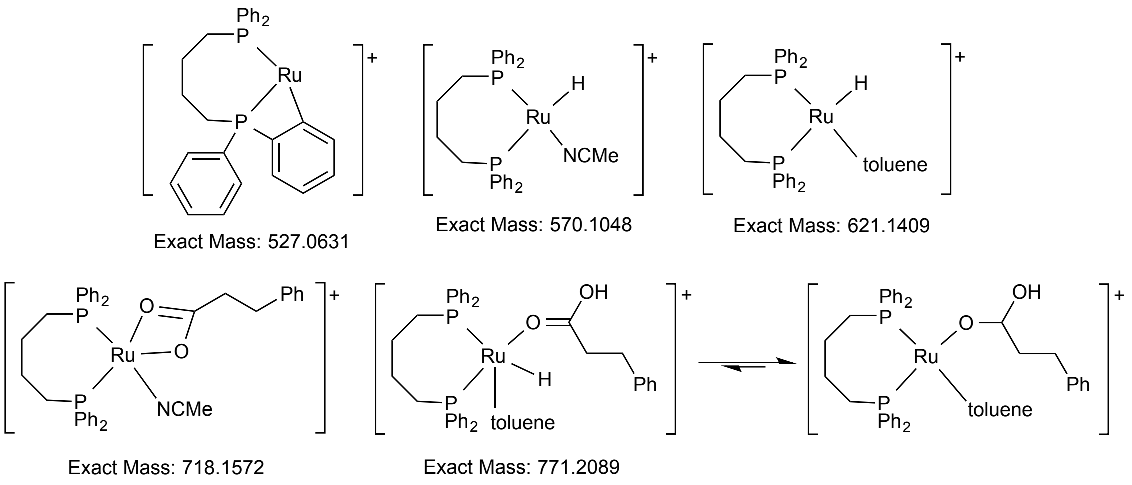

Supplementary Figure 8 | ESI-MS spectrum of a mixture of Ru species ([Ru]$_0$ = 10 mM, [CA-**a**]$_0$ = 333 mM, P_{H2} = 4 MPa, T = 160 °C, and t = 3 h).

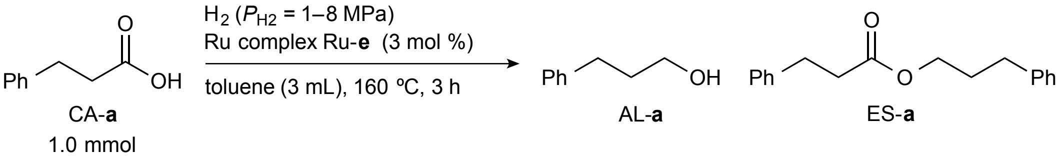

Enrty	H_2 (MPa)	^{1}H NMR yield (%)	
		AL-**a**	ES-**a**
1	1	12	1
2	2	25 ± 1	1 ± 0.5
3	3	34	2
4	4	39 ± 2	2 ± 0.5

Enrty	H_2 (MPa)	^{1}H NMR yield (%)	
		AL-**a**	ES-**a**
5	5	45	2
6	6	44 ± 3	2 ± 0
7	7	43	2
8	8	44 ± 3	2 ± 0

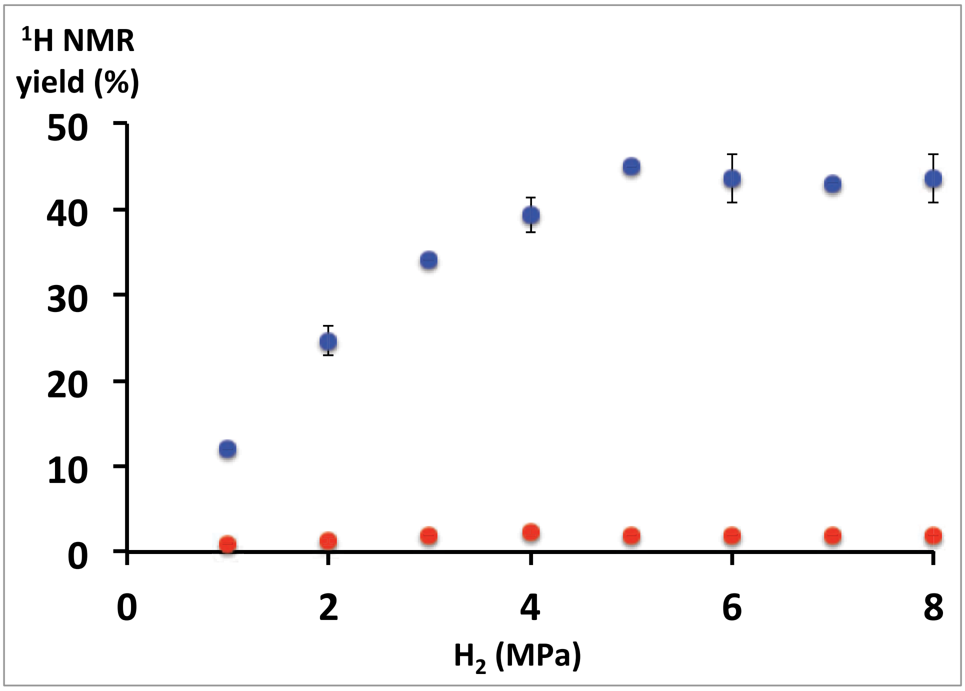

Supplementary Figure 9 | Hydrogen pressure dependency on yields of AL-**a** and ES-**a** using $Ru(OAc)_2[P(3,5\text{-xylyl})_3]_2$ (Ru-**e**).

Unless otherwise specified, the reactions were carried out with Ru-**e**:CA-**a** (mol %) = 3:100, T = 160 °C, and t = 3 h. ^{1}H NMR yields were determined based on the integral ratio of the signals of products and internal standard (mesitylene). The yields of products in entry 2, 4, 6 and 8 are average of three trials with calculated standard deviation. Blue and red circle represent the amount of AL-**a** (●) and ES-**a** (●●), respectively.

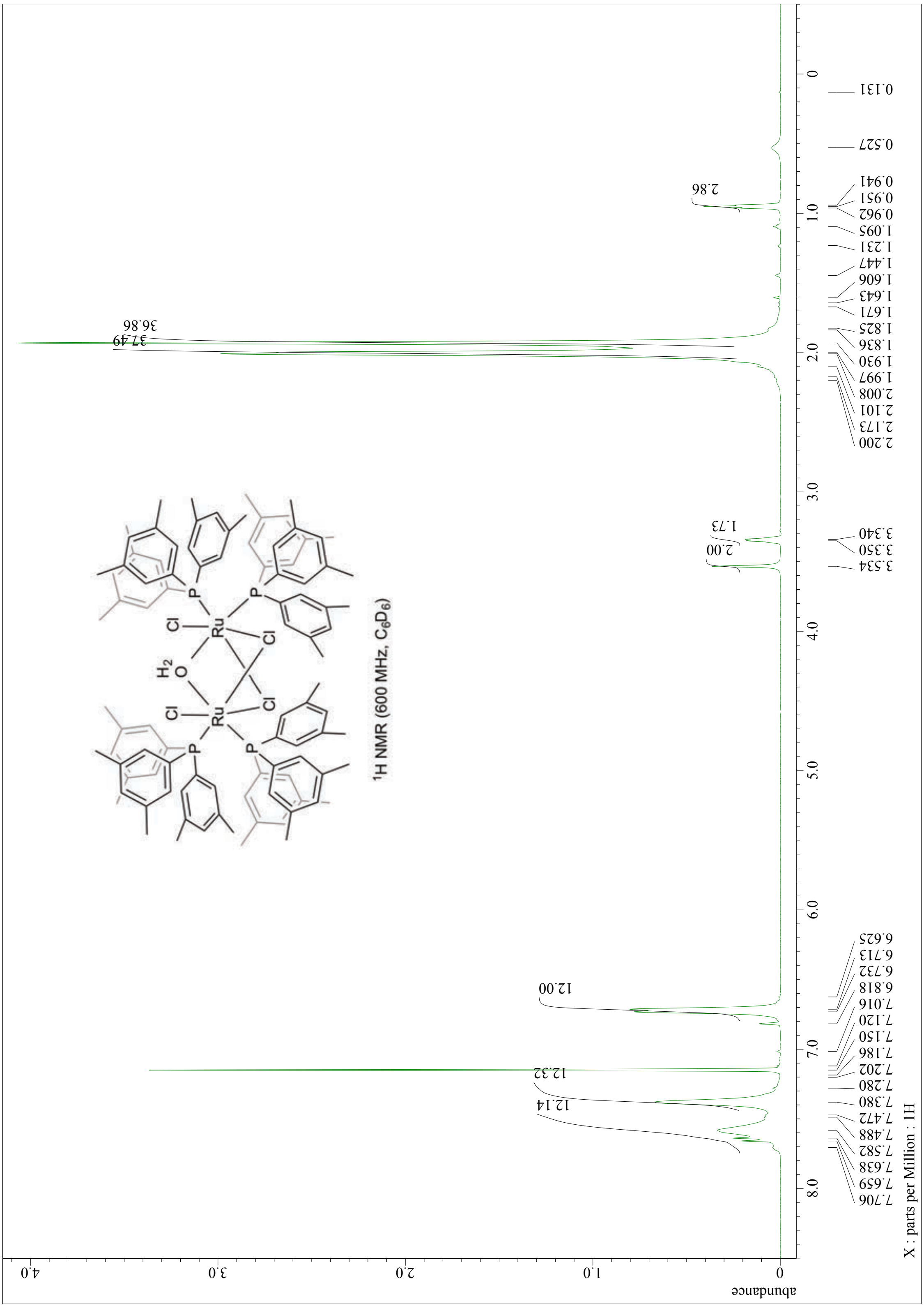

Supplementary Figure 10 | ^{1}H NMR spectrum of Ru complex Ru-**c** (Measurement temperature: 75 ºC).

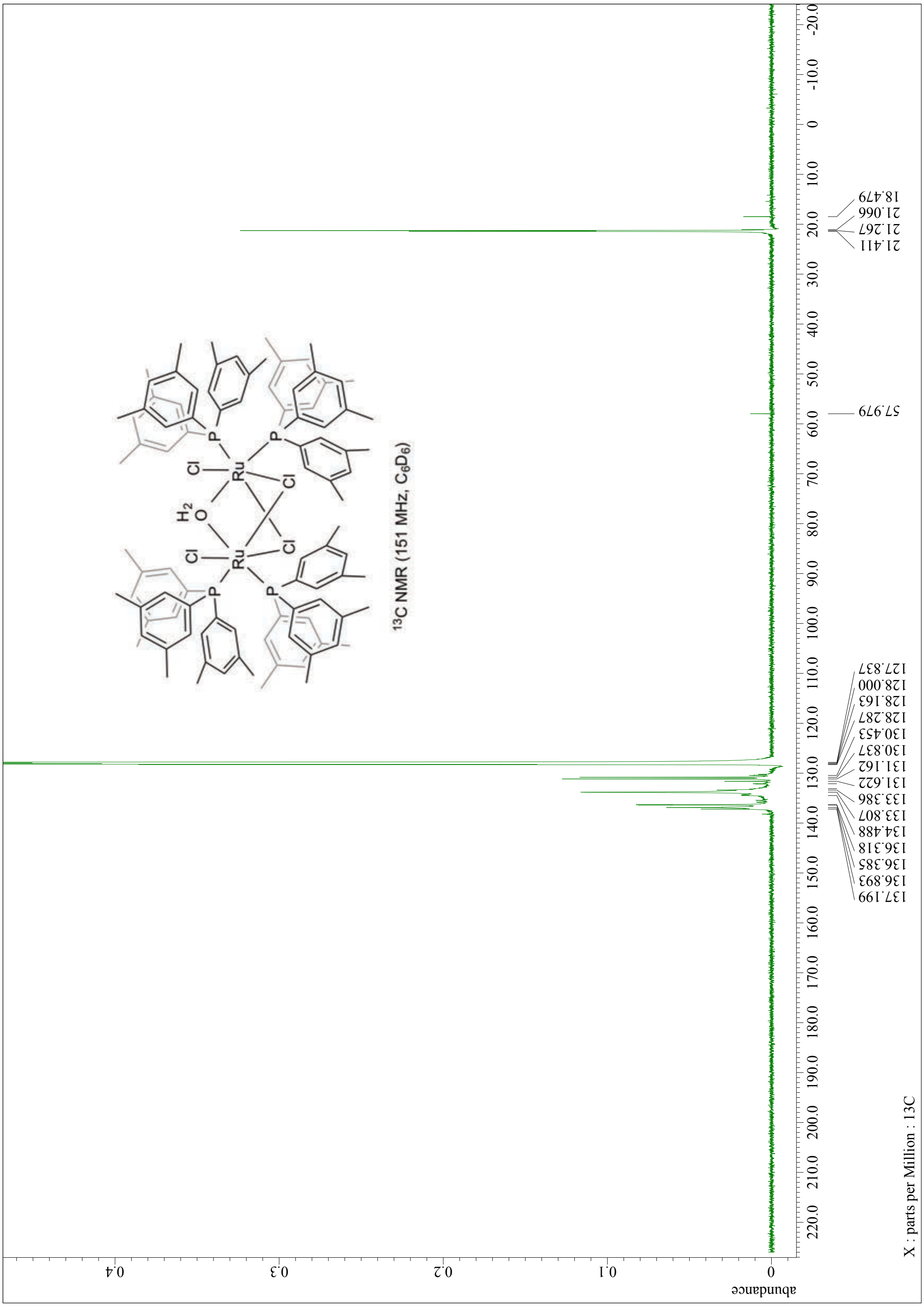

Supplementary Figure 11 | $^{13}C\{^1H\}$ NMR spectrum of Ru complex Ru-**c** (Measurement temperature: 75 ºC).

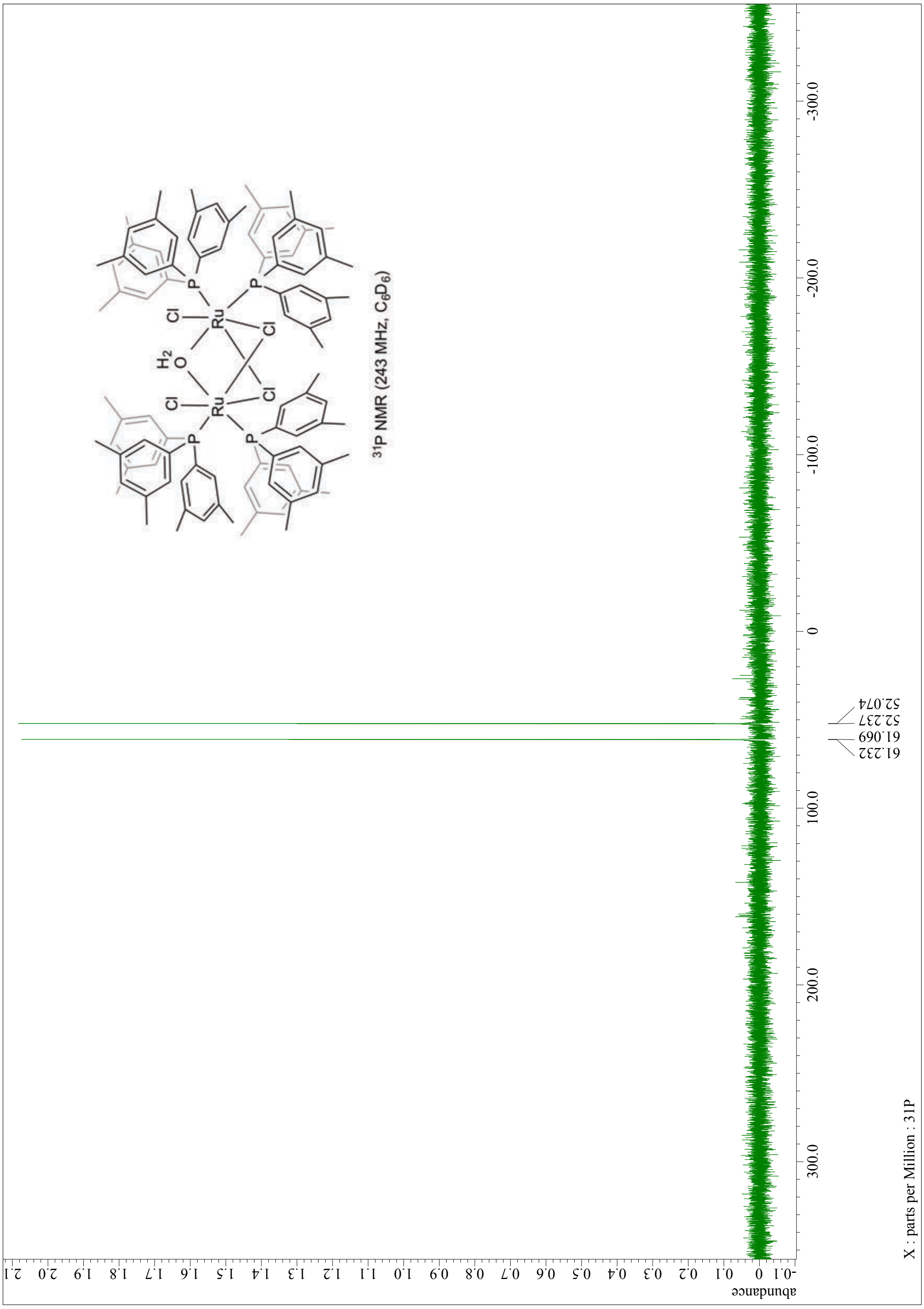

Supplementary Figure 12 | $^{31}P\{^{1}H\}$ NMR spectrum of Ru complex Ru-**c**.

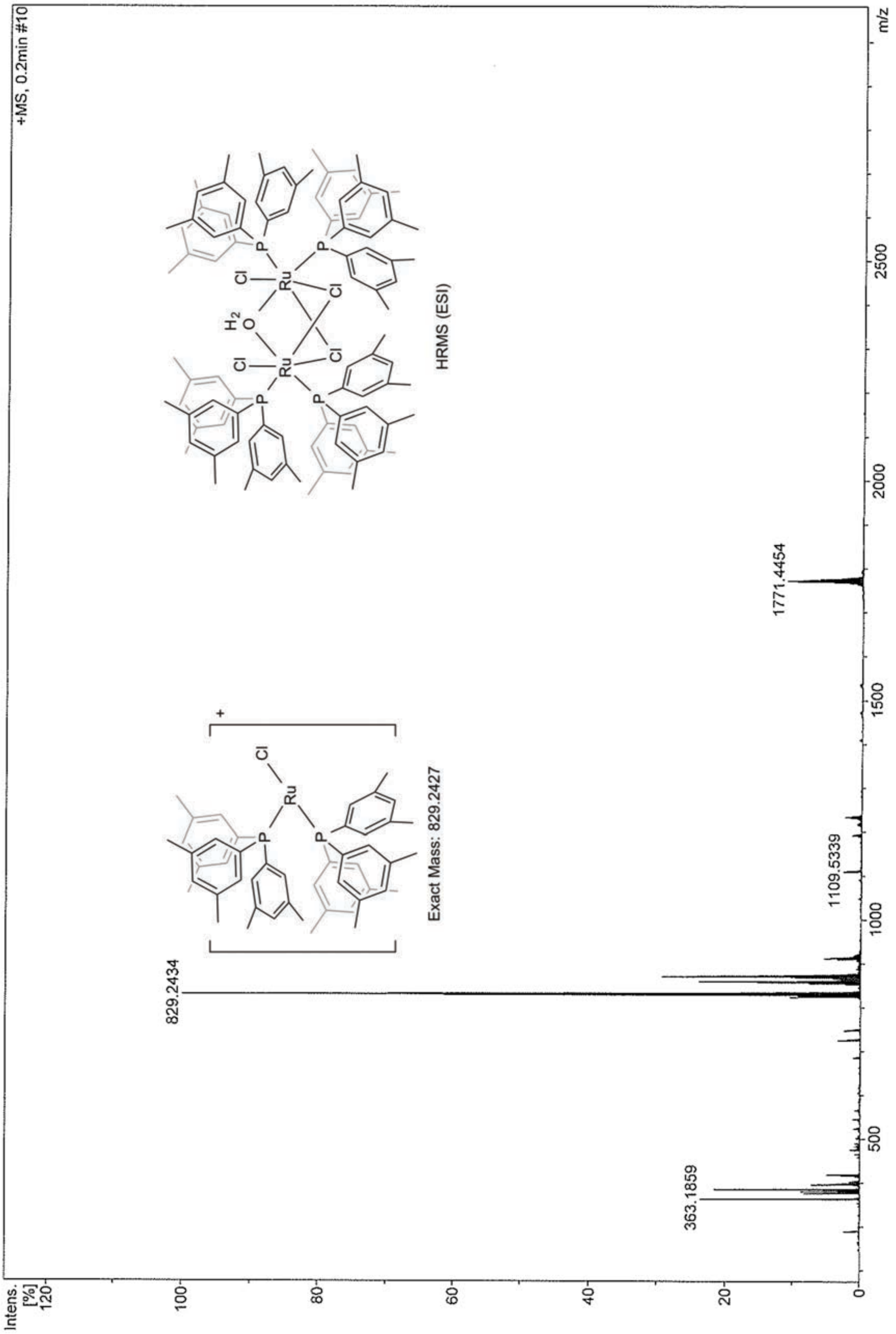

Supplementary Figure 13 | ESI-MS spectrum of Ru complex Ru-**c** (Sample preparation: Ru-**c** was dissolved in CH_2Cl_2 and diluted with acetonitrile).

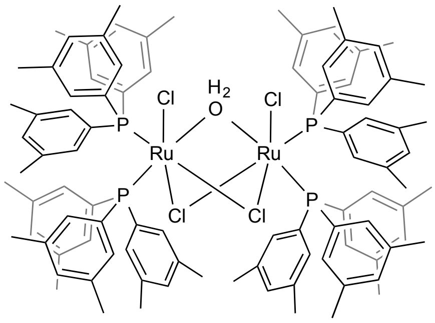

IR (KBr)

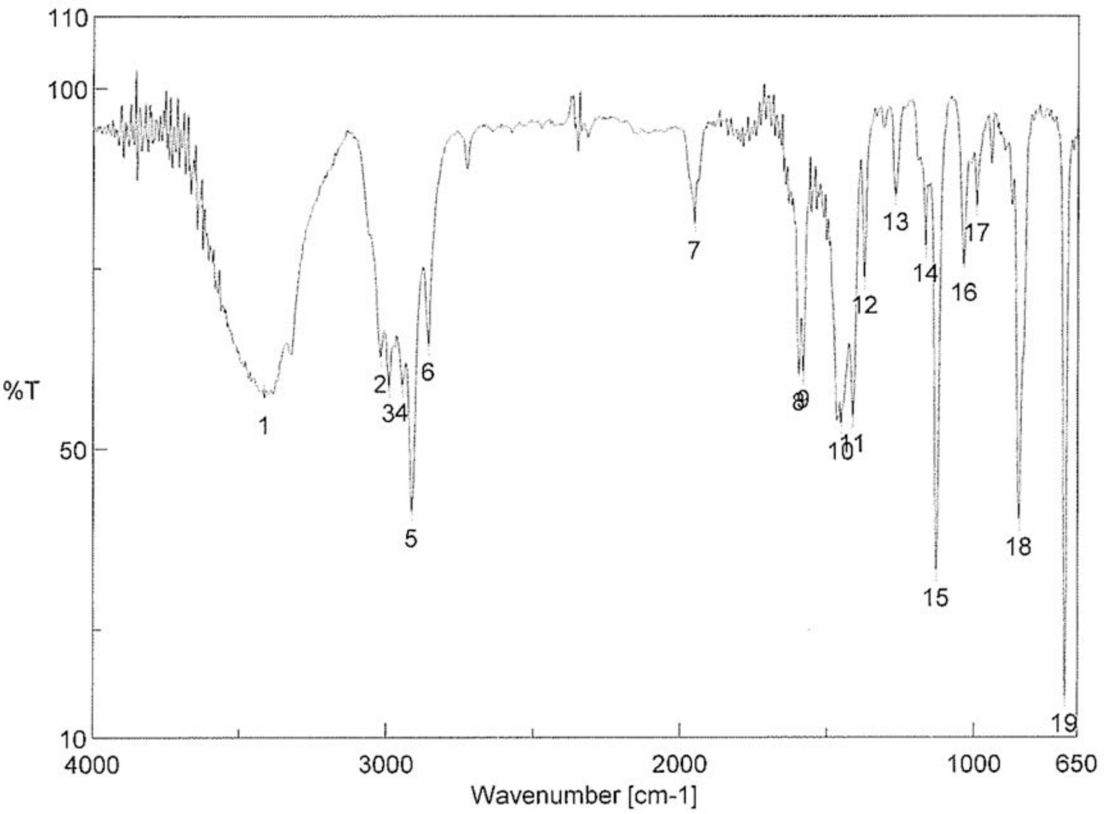

Supplementary Figure 14 | IR spectrum of Ru complex Ru-**c** (KBr).

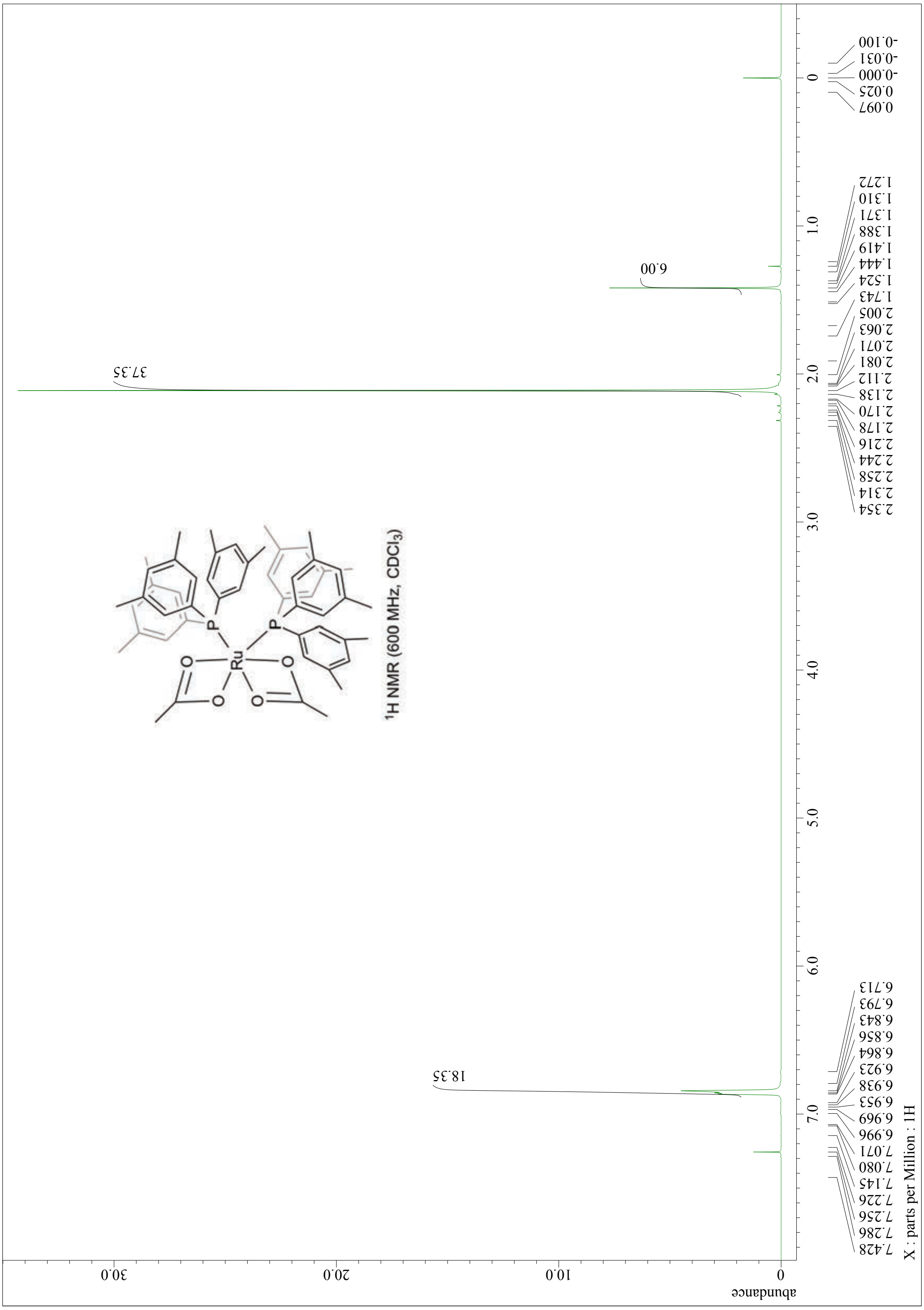

Supplementary Figure 15 | ^{1}H NMR spectrum of Ru complex Ru-**e**.

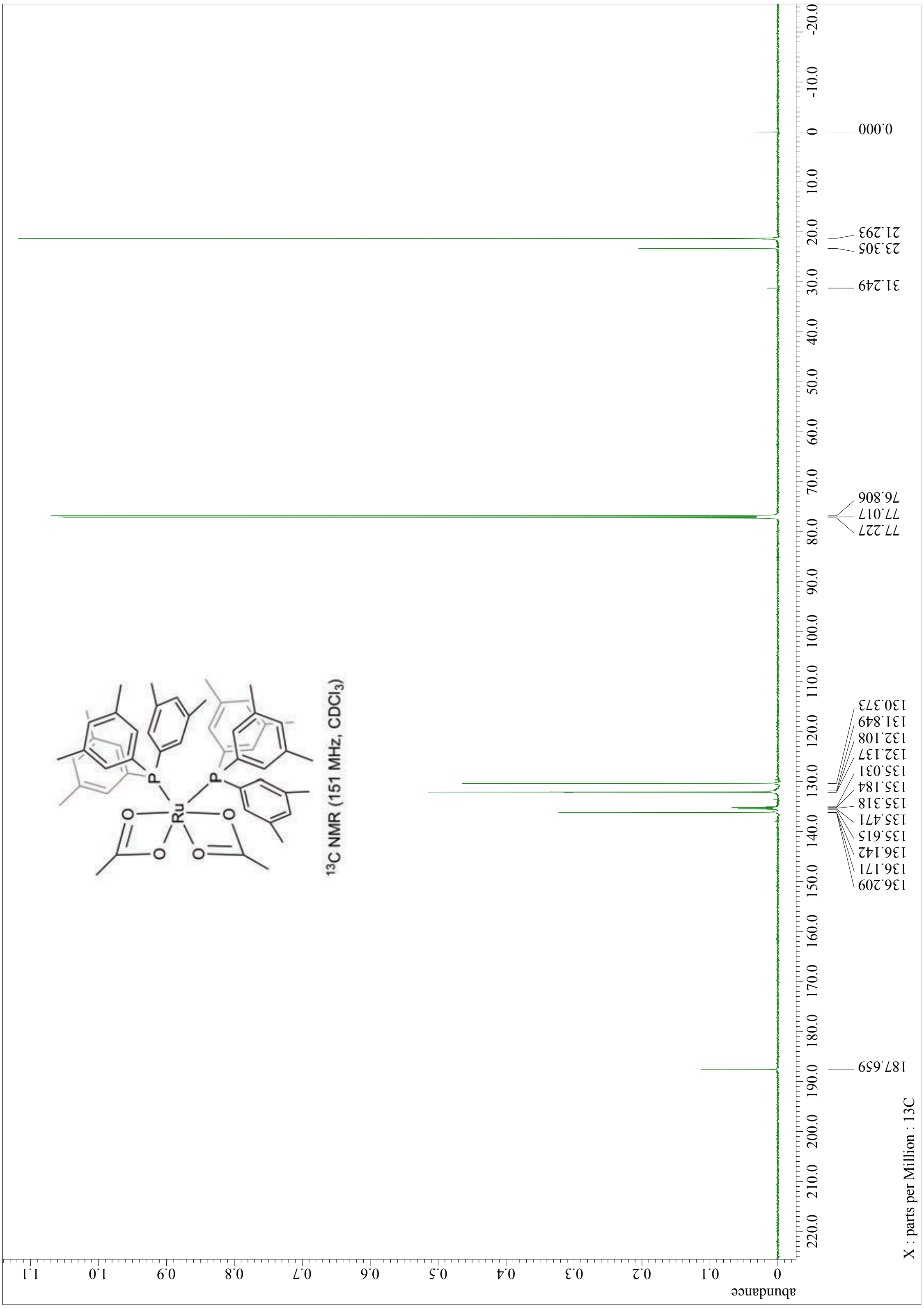

Supplementary Figure 16 | $^{13}C\{^{1}H\}$ NMR spectrum of Ru complex Ru-**e**.

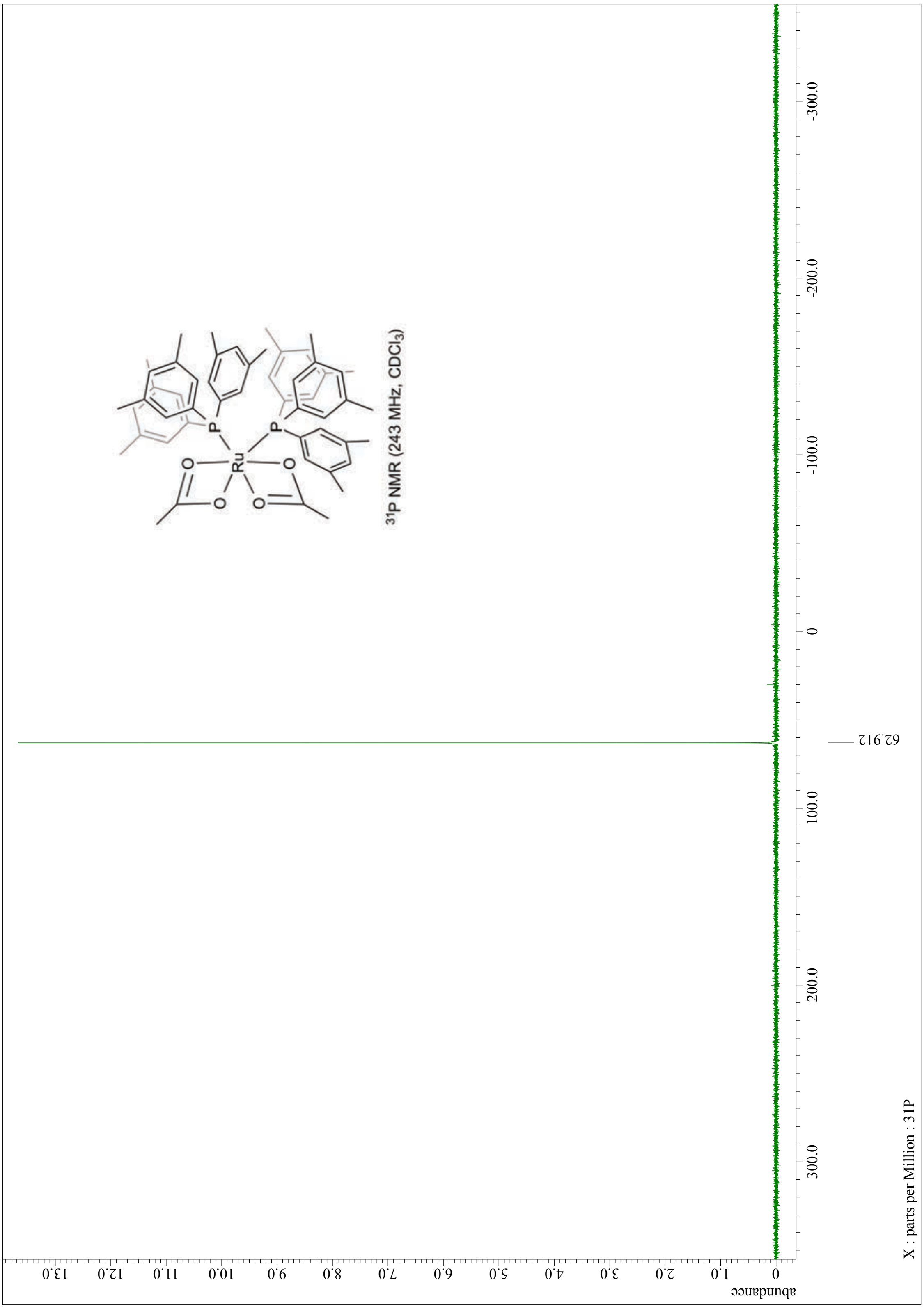

Supplementary Figure 17 | ^{31}P{^{1}H} NMR spectrum of Ru complex Ru-**e**.

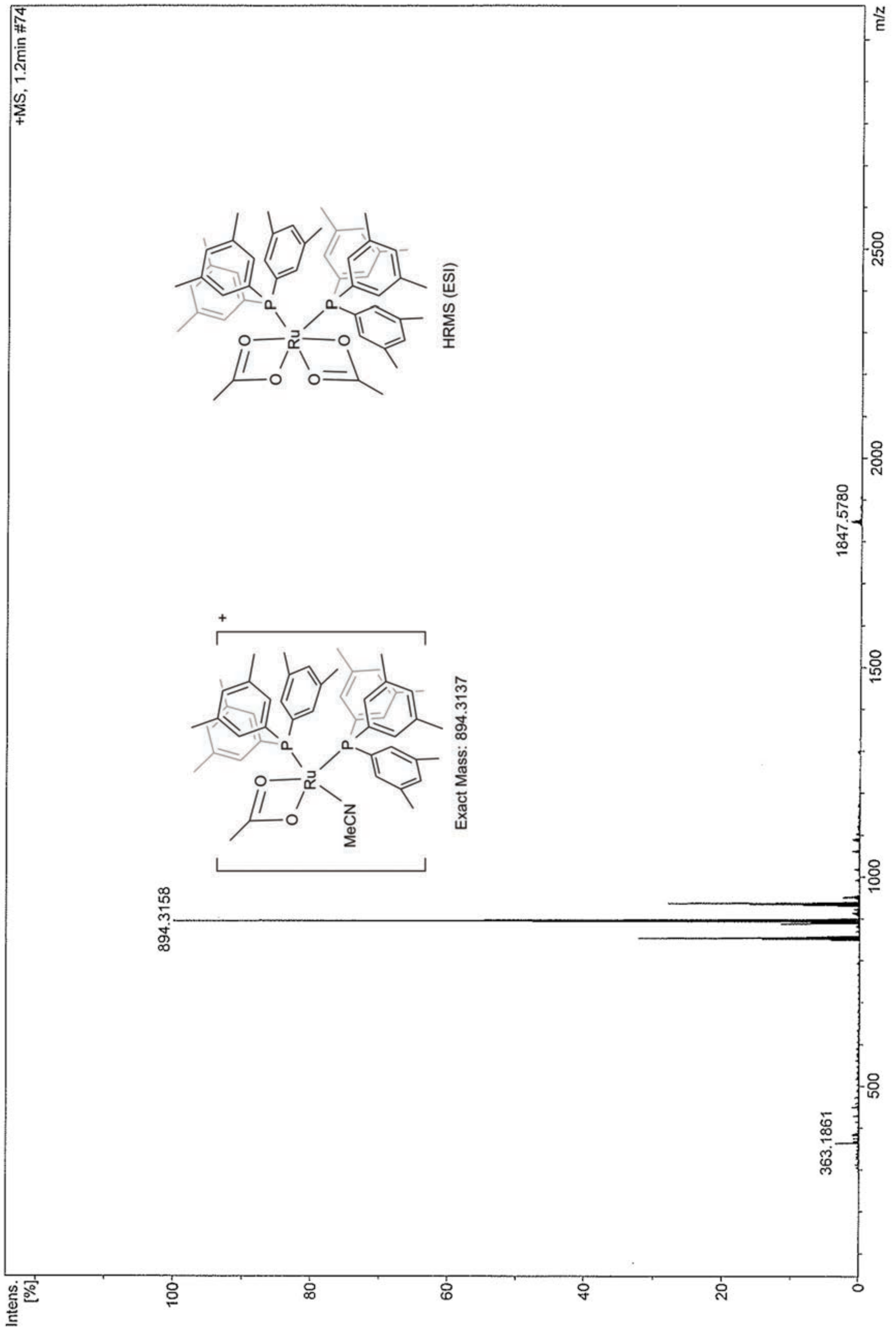

Supplementary Figure 18 | ESI-MS spectrum of Ru complex Ru-**e** (Sample preparation: Ru-**e** was dissolved in CH_2Cl_2 and diluted with acetonitrile).

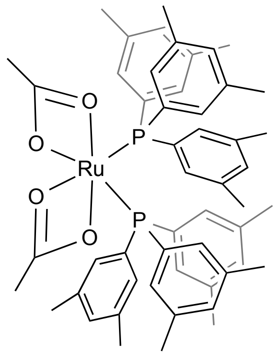

IR (KBr)

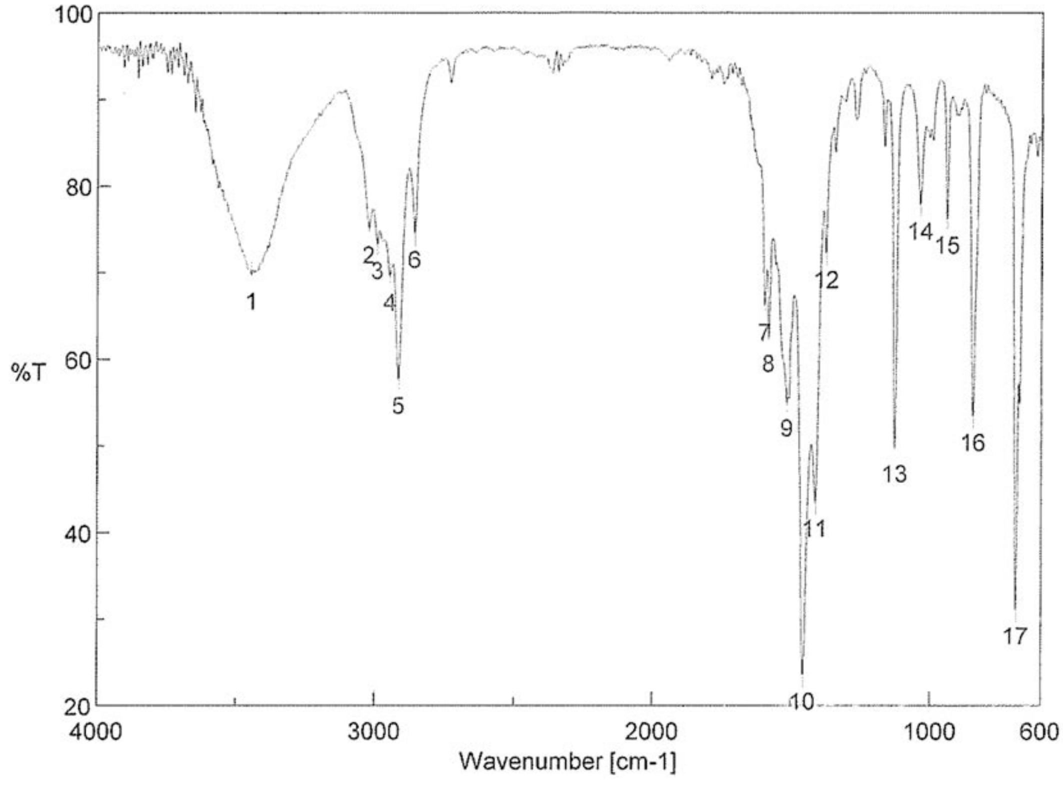

Supplementary Figure 19 | IR spectrum of Ru complex Ru-**e** (KBr) (O–H stretching was observed at 3447 cm^{-1} because of the hygroscopicity of KBr).

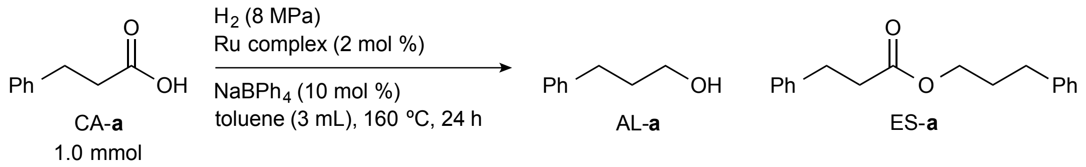

Entry	Ru complex	^{1}H NMR yield (%) AL-**a**	ES-**a**
1	$RuCl_2(PPh_3)_3$ (Ru-**a**)	58	16
2	$RuCl_2(PPh_3)_4$	8	9
3	$RuCl_2(CO)_2(PPh_3)_2$	2	6
4	$RuHCl(CO)(PPh_3)_3$	7	8
5	$RuCl(OAc)(PPh_3)_3$ (Ru-**b**)	55	17
6	$CpRuCl(PPh_3)_2$	3	5
7[a]	CpRuCl(dppm)	3	8
8	(*p*-cymene)$RuCl_2(PCy_3)$	trace	trace
9	*cis*-$RuCl_2(DMSO)_4$	2	3

Supplementary Table 1 | Ru complexes tested in the initial screening.
Unless otherwise specified, the reactions were carried out with
Ru complex:$NaBPh_4$:CA-**a** (mol %) = 2:10:100, P_{H2} = 8 MPa, T = 160 ºC, and t = 24 h.
^{1}H NMR yields were determined based on the integral ratio of the signals of products and internal standard (mesitylene).
[a] dppm = 1,1-bis(diphenylphosphino)methane.

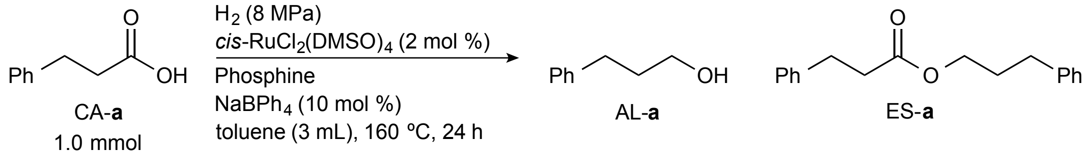

Entry	Phosphine (mol %)	Ru : Phosphine	^{1}H NMR yield (%) AL-**a**	ES-**a**
1	L-**a** (2 mol %)	1 : 1	3	6
**2**	L-**a (4 mol %)**	**1 : 2**	**31**	**15**
**3**	L-**a (6 mol %)**	**1 : 3**	**33**	**14**
4	L-**a** (8 mol %)	1 : 4	23	12
5	L-**a** (10 mol %)	1 : 5	7	10
6	L-**b** (6 mol %)	1 : 3	27	13
7	L-**c** (6 mol %)	1 : 3	39	15
8	L-**d** (6 mol %)	1 : 3	0	4
**9**	L-**e (6 mol %)**	**1 : 3**	**49**	**14**
10	L-**f** (6 mol %)	1 : 3	34	20
11	L-**g** (6 mol %)	1 : 3	5	9
12	L-**h** (6 mol %)	1 : 3	3	6
13	L-**i** (6 mol %)	1 : 3	5	7
14	L-**j** (6 mol %)	1 : 3	3	7
15	L-**k** (6 mol %)	1 : 3	4	7
16[a]	L-**l** (6 mol %)	1 : 3	trace	3
17	L-**m** (6 mol %)	1 : 3	1	7
18	L-**n** (2 mol %)	1 : 1	4	7
19	L-**o** (2 mol %)	1 : 1	5	7
20	L-**p** (2 mol %)	1 : 1	20	15
**21**	L-**q (2 mol %)**	**1 : 1**	**52**	**14**
22	L-**r** (2 mol %)	1 : 1	22	13
23	L-**s** (2 mol %)	1 : 1	48	18
24	L-**t** (2 mol %)	1 : 1	21	13

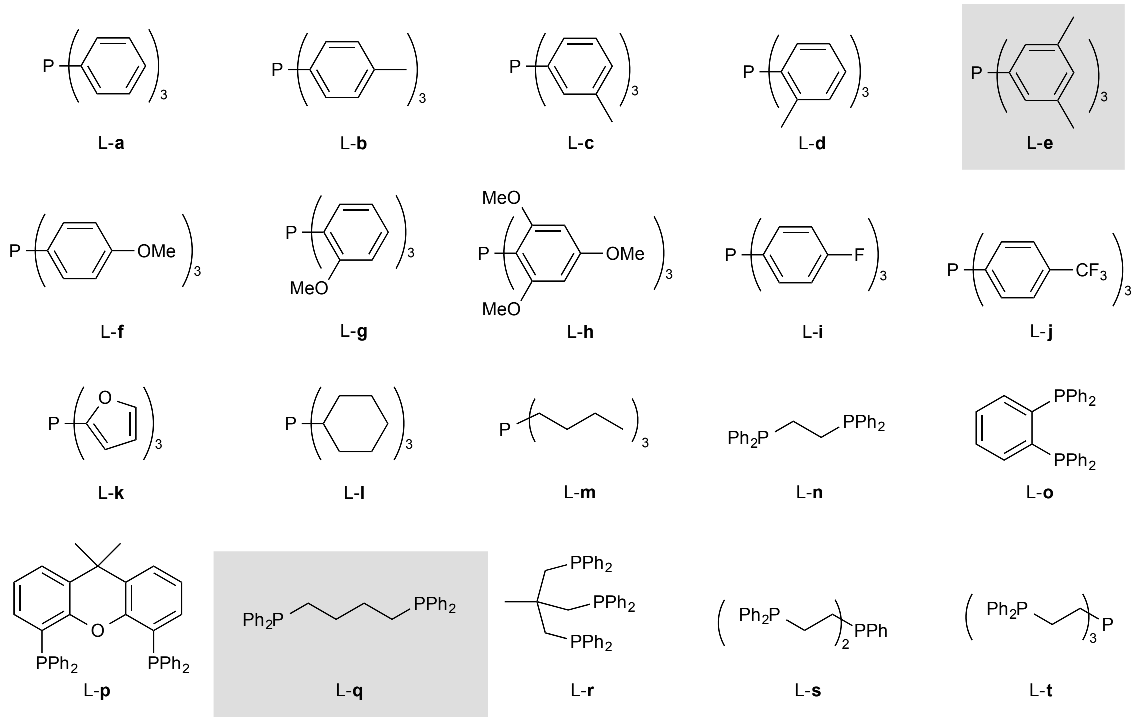

Supplementary Table 2 | Phosphine ligands screened with *cis*-$RuCl_2(DMSO)_4$ (2 mol %).

Unless otherwise specified, the reactions were carried out with *cis*-$RuCl_2(DMSO)_4$:$NaBPh_4$:CA-**a** (mol %) = 2:10:100, P_{H2} = 8 MPa, T = 160 ºC, and t = 24 h. ^{1}H NMR yields were determined based on the integral ratio of the signals of products and internal standard (mesitylene).

[a] 1 M toluene solution of L-**l** was used.

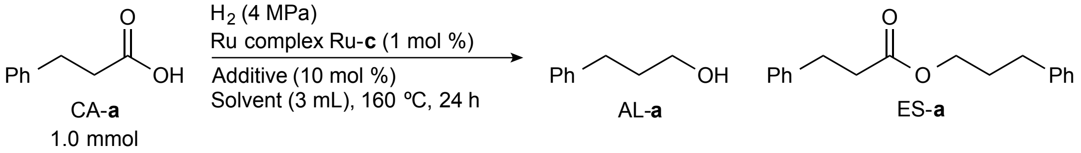

Entry	Additive	Solvent	^{1}H NMR yield (%)	
			AL-**a**	ES-**a**
1	—	toluene	trace	trace
2	NaOAc	toluene	62	15
3[a]	$LiBPh_4$	toluene	1	6
4	$NaBPh_4$	toluene	65	12
5	$KBPh_4$	toluene	63	12
6	$CsBPh_4$	toluene	60	12
7	$NaB(3,5\text{-}(CF_3)_2Ph)_4$	toluene	1	1
8	$NaBF_4$	toluene	trace	trace
9	$NaNTf_2$	toluene	trace	trace
10	NaOTs	toluene	trace	trace
11	NaOTf	toluene	trace	3
12[b]	NaH	toluene	58	14
13[c]	Na(acac)	toluene	64	14
14	NaOAc	CF_3-C_6H_5	48	18
15	NaOAc	1,4-dioxane	28	2
16	NaOAc	THF	35	2
17	NaOAc	*i*-PrOH	32	trace
18	NaOAc	*t*-BuOH	46	1

Supplementary Table 3 | Optimization of reaction conditions with Ru-**c**.

Unless otherwise specified, the reactions were carried out with Ru-**c**:additive:CA-**a** (mol %) = 1:10:100, P_{H2} = 4 MPa, T = 160 ºC, and t = 24 h. ^{1}H NMR yields were determined based on the integral ratio of the signals of products and internal standard (mesitylene).

[a] Lithium tetraphenylborate tris(1,2-dimethoxyethane) was used.

[b] NaH (55 % dispersion in paraffin liquid) was used.

[c] Na(acac)•xH_2O was used.

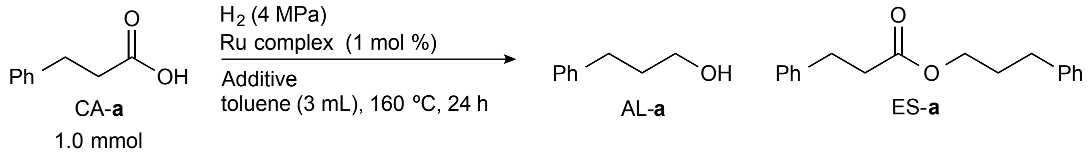

Entry	Ru complex	Additive	^{1}HNMR yield (%)	
			AL-**a**	ES-**a**
1	Ru-**c**	$NaBPh_4$ (10 mol %)	65	12
2	Ru-**c**	$NaBPh_4$ (10 mol %)<br>H_2O (0.5 mL)	10	7
3	Ru-**c**	NaOAc (10 mol %)	62	15
4	Ru-**c**	NaOAc (10 mol %)<br>H_2O (0.5 mL)	8	10
5[a]	Ru-**c**	Na(acac) (10 mol %)	64	14
6[a]	Ru-**c**	Na(acac) (10 mol %)<br>H_2O (0.5 mL)	10	7
7	Ru-**d**	$NaBPh_4$ (10 mol %)	18	12
8	Ru-**d**	$NaBPh_4$ (10 mol %)<br>H_2O (0.5 mL)	43	15
9	Ru-**d**	NaOAc (10 mol %)	54	15
10	Ru-**d**	NaOAc (10 mol %)<br>H_2O (0.5 mL)	35	16
11[a]	Ru-**d**	Na(acac) (10 mol %)	78	10
12[a]	Ru-**d**	Na(acac) (10 mol %)<br>H_2O (0.5 mL)	36	17

Supplementary Table 4 | Hydrogenation of 3-phenylpropionic acid (CA-**a**) in the presence of H_2O.

Unless otherwise specified, the reactions were carried out with Ru complex:Na salt: CA-**a** (mol %) = 1:10:100, P_{H2} = 4 MPa, T = 160 °C, and t = 24 h. ^{1}H NMR yields were determined based on the integral ratio of the signals of products and internal standard (mesitylene).

[a] Na(acac)•xH_2O was used.

Supplementary Method

1. General methods.

All experiments were performed under an inert gas atmosphere unless otherwise noted. ^{1}H NMR spectra were measured on JEOL ECA–600 (600 MHz), JEOL ECA–500 (500 MHz) at ambient temperature unless otherwise noted. Data were recorded as follows: chemical shift in ppm from internal tetramethylsilane (δ 0 ppm) or residual peak of C_6D_6 (δ 7.15 ppm), multiplicity (s = singlet, d = doublet, t = triplet, m = multiplet, bs = broad singlet), coupling constant (Hz), integration, and assignment. ^{13}C NMR spectra were measured on JEOL ECA–600 (150 MHz), JEOL ECA–500 (126 MHz) at ambient temperature. Chemical shifts were recorded in ppm from the solvent resonance employed as the internal standard (chloroform-*d* at 77.0 ppm or benzene-d_6 at 128.0 ppm or tetramethylsilane at 0 ppm). ^{31}P NMR spectra were measured on JEOL ECA–600 (243 MHz), JEOL ECA–500 (202 MHz) at ambient temperature. Chemical shifts (δ) were recorded in ppm from the solvent resonance employed as the external standard (phosphoric acid (85 wt % in H_2O) at 0.0 ppm). High–resolution mass spectra (HRMS) were obtained from BRUKER micrOTOF-QII (ESI), JEOL JMS-T100GCV (EI). For GC-MS analysis, Agilent GC-MS was used (GC: Agilent 6850 Network GC system or 7820A GC system; MS: Agilent 5975 series MSD or 5977E MSD; Column: J&W 19091S-433E (HP-5MS, 30 m × 0.250 mm × 0.25 μm); Carrier gas: Ar). IR spectra were obtained from JASCO FT/IR6100. For thin–layer chromatography (TLC) analysis throughout this work, Merck precoated TLC plates (silica gel 60 GF254 0.25 mm) were used. The products were purified by preparative column chromatography on silica gel 60 N (spherical, neutral) (40–100 μm; Kanto).

2. Materials.

4-(2-Thienyl)butyric acid (CA-**g**), $RuCl_2(PPh_3)_4$, CpRuCl(dppm), *cis*-$RuCl_2(DMSO)_4$, tris(4-methoxyphenyl)phosphine (L-**f**), tris(2,4,6-trimethoxyphenyl)phosphine (L-**h**), tricyclohexylphosphine (1 M in toluene) (L-**l**), 9,9-dimethyl-4,5-bis(diphenylphosphino)xanthene (Xantphos) (L-**p**), $NaBPh_4$, $KBPh_4$, $CsBPh_4$, $NaNTf_2$, NaOTs, NaOTf, *α*,*α*,*α*-trifluorotoluene and $(CH_3)_3COH$ (*t*-BuOH, anhydrous) were purchased from Aldrich. $RuCl_2(PPh_3)_3$ (Ru-**a**),

$RuCl_2(CO)_2(PPh_3)_2$, $RuHCl(CO)(PPh_3)_3$, $RuCl(OAc)(PPh_3)_3$ (Ru-**b**), (*p*-cymene)$RuCl_2(PCy_3)$, tri(*m*-tolyl)phosphine (L-**c**), tris(3,5-xylyl)phosphine (L-**e**), tris(*o*-methoxyphenyl)phosphine (L-**g**), tris(4-fluorophenyl)phosphine (L-**i**), 1,2-bis(diphenylphosphino)ethane (dppe) (L-**n**), 1,2-bis(diphenylphosphino)benzene (L-**o**), 1,1,1-tris(diphenylphosphinomethyl)ethane (triphos) (L-**r**), bis(2-diphenylphosphinoethyl)phenylphosphine (L-**s**), tris[2-(diphenylphosphino)ethyl]phosphine (L-**t**), $LiBPh_4•3(CH_3OCH_2CH_2OCH_3)$, $NaBF_4$, 1,4-dioxane (dehydrated), benzene (dehydrated), Tiglic acid (CA-**k**) and hexane (dehydrated) were purchased from Wako Pure Chemical industries, Ltd. *Trans*-Cinnamic acid (CA-**j**), $CpRuCl(PPh_3)_2$, triphenylphosphine (L-**a**), tri(*p*-tolyl)phosphine (L-**b**), tri-*n*-butylphosphine (L-**m**), NaOAc, NaH (55 % dispersion in paraffin liquid), toluene (dehydrated), THF (dehydrated), 2-propanol (dehydrated), ethanol, chloroform, diethyl ether (dehydrated), Celite 545, MeOH (dehydrated), acetone, ethyl acetate and mesitylene were purchased from Kanto Chemicals, Ltd. 3-Phenylpropionic acid (CA-**a**), stearic acid (CA-**b**), cyclohexanepropionic acid (CA-**c**), 1-adamantanecarboxylic acid (CA-**d**), phenoxyacetic acid (CA-**e**), monomethyl suberate (CA-**h**), 5-(benzoylamino)valeric acid (CA-**f**), 4-(trifluoromethyl)benzoic acid (CA-**n**), 4-*tert*-butylbenzoic acid (CA-**m**), tri(*o*-tolyl)phosphine (L-**d**), tri(2-furyl)phosphine (L-**k**), 1,4-bis(diphenylphosphino)butane (dppb) (L-**q**), 1,1,2,2-tetrachloroethane, 3-phenyl-1-propanol (AL-**a**), DL-2-Methyl-1-butanol (AL-**k**), 2-Methylbutyl DL-2-Methylbutyrate (ES-**k**) and anisole were purchased from TCI, Ltd. Benzoic acid (CA-**l**) was purchased from Nacalai. Tris(*p*-trifluoromethylphenyl)phosphine (L-**j**) was purchased from Strem. $Na(acac)•xH_2O$ was purchased from Alfa Aesar. $RuCl_3•nH_2O$ was purchased from Furuya Metals. $CDCl_3$ and C_6D_6 were purchased from Cambridge Isotope Laboratories, Inc. Hydrogen gas (H_2) was purchased from Alpha system (99.99 %). These chemicals were used without further purification. $NaB(3,5\text{-}(CF_3)_2C_6H_3)_4$,[1,2] $RuCl_2(PPh_3)(dppb)$,[3,4] and methyl-(*E*)-4-(2-carboxyvinyl)benzoate (CA-**i**)[5] were prepared according to the reported procedures.

3. Synthesis and characterizations

Preparation of [P(3,5-xylyl)$_3$]$_2$ClRu(μ-H$_2$O)(μ-Cl)$_2$RuCl[P(3,5-xylyl)$_3$]$_2$ (Ru-**c**)

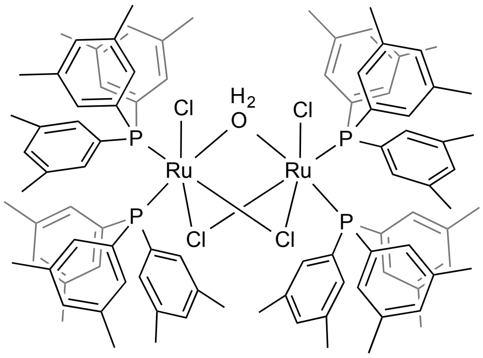

RuCl$_3$•nH$_2$O (containing Ru in 41.91 wt %) (241.7 mg, 1.0 mmol), degassed and Ar-purged ethanol (20 mL), and magnetic stirring bar were placed in a vessel equipped with a Young's stopcock (75 mL) under Ar gas atmosphere. The mixture was stirred at 90 ºC for 15 min and cooled to room temperature. To this reaction mixture was added tris(3,5-xylyl)phosphine (2.08 g, 6.0 mmol) and degassed ethanol (3 mL), and heated at 90 ºC for 19 h without stirring to afford reddish brown crystal. The crystal was collected by filtration under Ar gas atmosphere before the temperature of the mother liquid was cooled to room temperature, washed with degassed chloroform (3×20 mL) and degassed diethyl ether (3×10 mL), and dried *in vacuo* to obtain desired Ru complex Ru-**c** (721.8 mg, 0.41 mmol, 83 %). Ethanol is contained in a crystal structure.

The ^{1}H and ^{13}C{^{1}H} NMR of Ru-**c** measured at 25 ºC gave a set of complicated broad peaks, so that 75 °C was used instead.

^{1}H NMR (600 MHz, C$_6$D$_6$, 75 ºC): δ 7.58 (bs, 12H, 12Me$_2$C$_6$H$_2$*H*), 7.38 (bs, 12H, 12Me$_2$C$_6$H$_2$*H*), 6.80–6.66 (m, 12H, 12Me$_2$C$_6$H$_2$*H*), 3.53 (bs, 2H, *H*$_2$O), 2.01 (bs, 36H, 12C*H*$_3$), 1.93 (bs, 36H, 12C*H*$_3$); ^{13}C{^{1}H} NMR (151 MHz, C$_6$D$_6$, 75 ºC): δ 137.5–135.2 (m, 36C), 134.8–132.3 (m, 24C), 131.8–130.0(m, 12C), 21.4 (12C), 21.3 (12C); ^{31}P{^{1}H} NMR (243 MHz, C$_6$D$_6$): δ 61.2 (d, $^2J_{PP}$ = 39.5 Hz), 52.2 (d, $^2J_{PP}$ = 39.5 Hz); IR (KBr): 3417 (m), 3021 (m), 2993 (m), 2948 (m), 2916 (s), 2859 (m), 1955 (w), 1598 (m), 1582 (m), 1455 (m), 1415 (m), 1375 (w), 1271 (w), 1169 (w), 1131 (s), 1038 (w), 994 (w), 849 (m), 694 (s) cm^{-1}; HRMS (ESI) (*m/z*): [Ru[P(3,5-xylyl)$_3$]$_2$Cl]$^+$ calcd. for C$_{48}$H$_{54}$ClP$_2$Ru, 829.2427; found, 829.2434; analysis (calcd., found for C$_{96}$H$_{110}$Cl$_4$OP$_4$Ru$_2$•C$_2$H$_6$O): C (65.62, 65.69) H (6.52, H 6.45).

Preparation of $Ru(OAc)_2[P(3,5\text{-xylyl})_3]_2$ (Ru-e)

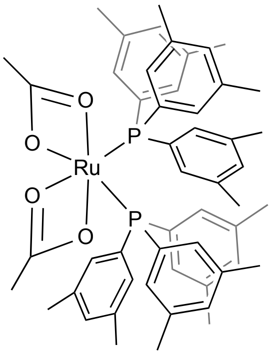

Ru complex Ru-**c** (Be advised to grind into as small particles as possible) (174.4 mg, 0.10 mmol), NaOAc (163.3 mg, 2.0 mmol), degassed and Ar-purged *t*-BuOH (25 mL), and magnetic stirring bar were placed in a vessel equipped with a Young's stopcock (75 mL) under Ar gas atmosphere. The mixture was stirred at 90 ºC for 2 h and cooled to room temperature. The mixture was filtered through a pad of Celite to remove a white residue, and washed with a small amount of *t*-BuOH under Ar gas atmosphere. The solution was concentrated under reduced pressure until orange precipitation was generated. The slurry was stirred and heated at 90 ºC using an oil bath until the orange precipitate was redissolved, and then stopped heating the oil to give orange crystal. After 14 h, the orange crystal was collected by filtration, washed with H_2O and *t*-BuOH, and dried *in vacuo* to obtain desired Ru complex Ru-**e** (90.1 mg, 0.099 mmol, 49 %).

^{1}H NMR (600 MHz, $CDCl_3$): δ 6.89–6.80 (m, 18H, $6Me_2C_6H_3$), 2.11 (s, 36H, $6(CH_3)_2Ph$), 1.42 (s, 6H, $2CH_3COO$); $^{13}C\{^1H\}$ NMR (151 MHz, $CDCl_3$): δ 187.7 (2C), 136.2 (t, J_{PC} = 4.33 Hz, 12C), 135.7–134.9 (m, 6C), 132.1 (t, J_{PC} = 4.34 Hz, 12C), 130.4 (6C), 23.3 (2C), 21.3 (12C); $^{31}P\{^1H\}$ NMR (243 MHz , $CDCl_3$): δ 62.9; HRMS (ESI) (*m/z*): $[Ru[P(3,5\text{-xylyl})_3]_2(OAc)(CH_3CN)]^+$ calcd. for $C_{52}H_{60}NO_2P_2Ru$, 894.3137; found, 894.3158; IR (KBr): 3447 (w), 3023 (w), 2992 (w), 2947 (w), 2916 (m), 2858 (w), 1599 (w), 1583 (w), 1517 (m, κ^2-OCO_{asym}),[6,7] 1458 (s, κ^2-OCO_{sym}),[6,7] 1414 (m), 1376 (w), 1129 (s), 1038 (w), 943 (w), 848 (m), 691 (s) cm^{-1}; analysis (calcd., found for $C_{52}H_{60}O_4P_2Ru$): C (68.48, 6.63) H (68.48, H 6.61).

Preparation of (dppb)ClRu(μ-H_2O)(μ-Cl)$_2$RuCl(dppb) (Ru-**d**)

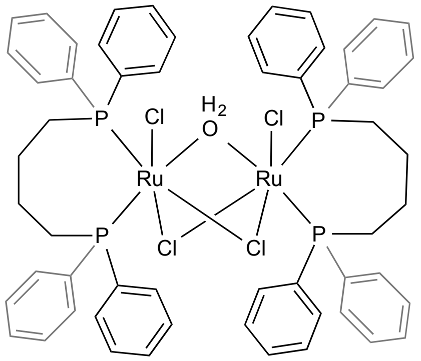

The complex Ru-**d** was prepared by minor modifications of the literature procedure[8] as shown below.

$RuCl_2(PPh_3)$(dppb) (420.1 mg, 0.49 mmol), benzene (20 mL) and magnetic stirring bar were placed in a vessel equipped with a Young's stopcock (100 mL) under Ar gas atmosphere, and the mixture was stirred at 80 ºC for 10 min. To this mixture was added H_2O (15 mL) and heated at 80 ºC for 1 h. The organic phase became dark orange. After cooling to room temperature, hexane/ethanol (3/1 v/v) was layered on the organic layer (ca. 1~2 mm), and then hexane (50 mL) was layered on the mixture. After very slow shaking to vanish the clear interface between dark orange layer and hexane layer, the mixture was kept on a lab bench at room temperature for 3 days to give reddish brown crystal. The crystal was collected by filtration, washed with MeOH and cold acetone (0 ºC), and dried *in vacuo* to obtain desired Ru complex Ru-**d** (193.4 mg, 0.16 mmol, 64 %).

^{1}H NMR (600 MHz, $CDCl_3$): δ 7.88–7.79 (m, 4H), 7.60–7.48 (m, 8H), 7.40–7.10 (m, 28H), 3.40 (bs, 2H), 2.51 (bs, 2H), 2.46–2.34(m, 2H), 2.34–2.20 (m, 2H), 1.95 (bs, 2H), 1.85–1.65 (m, 2H), 1.41 (bs, 2H), 1.35–1.20 (m, 4H); ^{31}P{^{1}H} NMR (243 MHz, $CDCl_3$): δ 64.2 (d, $^{2}J_{PP}$ = 46.0 Hz), 55.0 (d, $^{2}J_{PP}$ = 46.0 Hz); HRMS (ESI) (*m/z*): [Ru(dppb)Cl(CH_3CN)]$^+$ calcd. for $C_{30}H_{31}ClNP_2Ru$, 604.0658; found, 604.0668.

The spectral data were almost consistent with the reported values.[8] The single crystal structure of Ru-**d** has been also reported, but analyzed again this time via the X-ray diffraction study, and was consistent with the reported structure.[8]

Preparation of $Ru(OAc)_2$(dppb) (Ru-**f**)

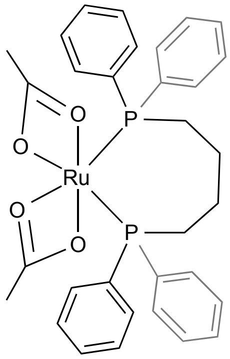

The complex Ru-**f** was synthesized using two different methods. The one is described in the reported procedure.[6]

Ru complex Ru-**d** (242.8 mg, 0.20 mmol), NaOAc (329.0 mg, 4.0 mmol), degassed and Ar-purged *t*-BuOH (25 mL), and magnetic stirring bar were placed in a vessel equipped with a Young's stopcock (75 mL) under Ar gas atmosphere. The mixture was stirred at 90 ºC for 5 h and cooled to room temperature. The reaction vessel was kept on lab bench at room temperature for about 6 h to generate a yellow precipitate. The precipitate was collected by filtration, washed with H_2O, *t*-BuOH and hexane, and then, dried *in vacuo* to obtain desired Ru complex Ru-**f** as yellow solid (128.8 mg, 0.20 mmol, 50 %).

^{1}H NMR (600 MHz, $CDCl_3$): δ 7.60–7.25 (m, 20H, 2P$(C_6H_5)_2$), 2.51 (bs, 4H, 2CH_2PPh), 1.68 (bs, 4H, 2CH_2), 1.49 (s, 6H, 2CH_3COO); ^{13}C{^{1}H} NMR (149 MHz, $CDCl_3$): δ 188.2 (2C), 132.5 (bs, 8C), 129.2 (bs, 4C), 128.0 (bs, 12C), 28.9 (d, J_{PC} = 15.8 Hz), 28.8 (d, J_{PC} = 14.3 Hz), 23.8 (2C), 23.7 (2C); ^{31}P{^{1}H} NMR (243 MHz, $CDCl_3$): δ 63.6; HRMS (ESI) (*m/z*): $[Ru(dppb)(OAc)(CH_3CN)]^+$ calcd. for $C_{32}H_{34}NO_2P_2Ru$, 628.1103; found, 628.1100; IR (KBr): 3424 (m), 3056 (m), 2926 (m), 2855 (w), 1519 (s), 1484 (m), 1455 (s), 1434 (s), 1411 (s), 1343 (w), 1188 (w), 1158 (w), 1128 (w), 1096 (m), 1027 (w), 999 (w), 942 (w), 906 (m), 857 (w), 814 (m), 785 (w), 743 (m), 697 (s) cm^{-1}.

The spectral data were almost consistent with the reported values other than ^{31}P{^{1}H} NMR (^{31}P{^{1}H} NMR ($CDCl_3$) δ 61.37).[6]

New ester compounds obtained from hydrogenation reactions

The structures of known esters ES-**a**, ES-**b**, ES-**d**, ES-**e** and ES-**k**–**n** were determined using ESI-MS, GC-MS (EI) and the ^{1}H NMR signals (singlet or triplet or multiplet) of -CO_2CH_2- at around δ 4.0 ppm, which indicate the existence of the ester functionality.

3-cyclohexylpropyl 3-cyclohexylpropanoate (ES-**c)**

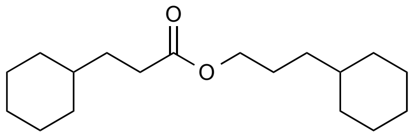

The reaction mixture obtained after hydrogenation was purified by column chromatography on silica gel (eluent; hexane/AcOEt = 10/1) giving ES-**c**.

TLC (hexane:AcOEt, 10:1 v/v): R_f = 0.66; ^{1}H NMR (600 MHz, $CDCl_3$): δ 4.04 (t, 2H, J = 6.9 Hz, CH_2OCO), 2.30 (t, 2H, J = 7.6 Hz, CH_2COO), 1.73–1.59 (m, 12H), 1.55–1.49 (m, 2H), 1.27–1.09 (m, 10H), 0.94–0.83 (m, 4H); ^{13}C{^{1}H} NMR (151 MHz , $CDCl_3$): δ 174.2, 64.7, 37.3, 37.2, 33.5, 33.3, 32.9, 32.4, 32.0, 26.6, 26.5, 26.3, 26.2, 26.0; HRMS (ESI) (*m/z*): $[M+Na]^+$ calcd. for $C_{18}H_{32}O_2Na$, 303.2295; found, 303.2285.

5-benzamidopentyl 5-benzamidopentanoate (ES-**f)**

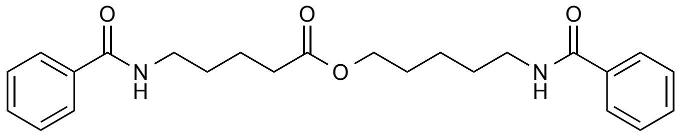

The reaction mixture obtained after hydrogenation was purified by column chromatography on silica gel (eluent; hexane/AcOEt = 1/2), giving ES-**f**.

TLC (hexane:AcOEt, 1:2 v/v): R_f = 0.33; ^{1}H NMR (600 MHz, $CDCl_3$): δ 7.80–7.75 (m, 4H), 7.49–7.45 (m, 2H), 7.42–7.37 (m, 4H), 6.65 (bs, 1H), 6.54 (bs, 1H), 4.09 (t, 2H, J = 6.1 Hz), 3.48–3.40 (m, 4H), 2.35 (t, 2H, J = 6.8 Hz), 1.75–1.60 (m, 8H), 1.48–1.40 (m, 2H); ^{13}C{^{1}H} NMR (149 MHz , $CDCl_3$): δ 173.6, 167.61, 167.58, 134.7, 134.6, 131.3 (2C), 128.4 (2C), 126.88, 126.86, 64.1, 39.9, 39.5, 33.8, 29.2, 29.0, 28.2, 23.4, 22.2; HRMS (ESI) (*m/z*): $[M+Na]^+$ calcd. for $C_{24}H_{30}N_2NaO_4$, 433.2098; found, 433.2068.

4-(thiophen-2-yl)butyl 4-(thiophen-2-yl)butanoate (ES-**g**)

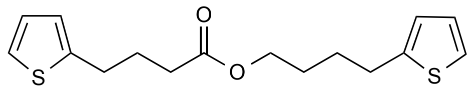

The reaction mixture obtained after hydrogenation was purified by column chromatography on silica gel (eluent; hexane/AcOEt = 20/1), giving ES-**g**.

TLC (hexane:AcOEt, 20:1 v/v): $R_f = 0.20$; ^{1}H NMR (600 MHz , $CDCl_3$): δ 7.13–7.10 (m, 2H), 6.94–6.90 (m, 2H), 6.81–6.77 (m, 2H), 4.10 (t, 2H, J = 6.2 Hz), 2.92–2.82 (m, 4H), 2.36 (t, 2H, J = 7.6 Hz), 2.04–1.97 (m, 2H), 1.78–1.68 (m, 4H); $^{13}C\{^1H\}$ NMR (151 MHz, $CDCl_3$): δ 173.3, 144.8, 144.1, 126.8, 126.7, 124.5, 124.2, 123.2, 123.0, 64.1, 33.4, 29.4, 29.1, 28.1, 28.0, 26.8; HRMS (EI) (*m/z*): $[M]^+$ calcd. for $C_{16}H_{20}O_2S_2$, 308.0899; found, 308.0894.

1-(8-methoxy-8-oxooctyl) 8-methyl octanedioate (ES-**h**)

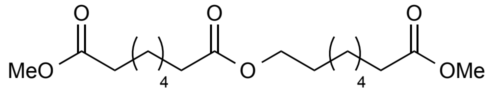

The reaction mixture obtained after hydrogenation was purified by column chromatography on silica gel (eluent; hexane/AcOEt = 1/1), giving ES-**h**.

TLC (hexane:AcOEt, 1:1 v/v): $R_f = 0.52$; ^{1}H NMR (600 MHz, $CDCl_3$): δ 4.05 (t, 2H, J = 6.8 Hz), 3.67 (s, 6H), 2.34–2.26 (m, 6H), 1.67–1.58 (m, 8H), 1.38–1.30 (m, 10H); $^{13}C\{^1H\}$ NMR (151 MHz, $CDCl_3$): δ 174.2, 174.1, 173.8, 64.3, 51.4 (2C), 34.2, 33.99, 33.95, 29.0, 28.8, 28.7 (2C), 28.5, 25.7, 24.79, 24.75, 24.71; HRMS (ESI) (*m/z*): $[M+Na]^+$ calcd. for $C_{18}H_{32}O_6Na$, 367.2091; found, 367.2083.

methyl 4-(3-((3-(4-(methoxycarbonyl)phenyl)propanoyl)oxy)propyl)benzoate (ES-**i**)

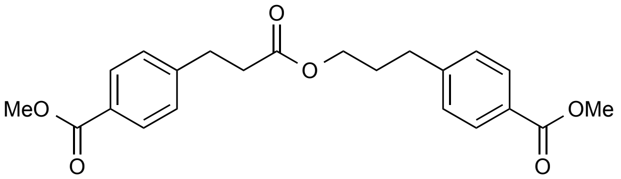

The reaction mixture obtained after hydrogenation was fractionated by column chromatography on silica gel (eluent; hexane/AcOEt = 2/1), giving ES-**i**.

TLC (hexane;AcOEt, 2:1 v/v): $R_f = 0.39$; ^{1}H NMR (600 MHz, $CDCl_3$): δ 7.96 (d, 2H, J = 8.3 Hz), 7.95 (d, 2H, J = 7.6 Hz), 7.27 (d, 2H, J = 8.2 Hz), 7.20 (d, 2H, J = 8.2 Hz), 4.08 (t, 2H, J = 6.2 Hz), 3.902 (s, 3H), 3.896 (s, 3H), 3.0 (t, 2H, J = 7.6 Hz),

2.69–2.62 (m, 4H), 1.96–1.90 (m, 2H); $^{13}C\{^{1}H\}$ NMR (151 MHz, $CDCl_3$): δ 172.5, 167.0, 166.9, 146.6, 145.8, 129.84, 129.78, 128.4, 128.3 (2C), 128.1, 63.7, 52.0 (2C), 35.3, 32.2, 30.9, 29.8; HRMS (ESI) (*m/z*): $[M+Na]^+$ calcd. for $C_{22}H_{24}O_6Na$, 407.1465; found, 407.1445.

4. Hydrogenation

Representative procedure for the hydrogenation of carboxylic acids: The reaction of 3-phenylpropionic acid (CA-a) (Table 1, Entry 1)

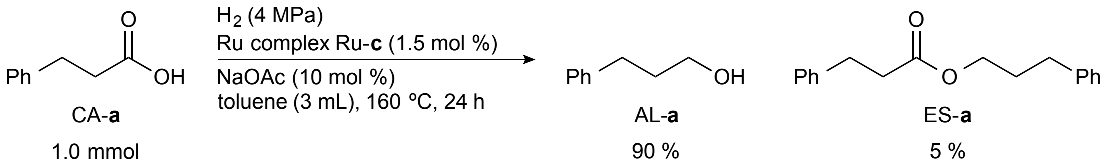

3-Phenylpropionic acid (CA-**a**) (150.2 mg, 1.0 mmol), Ru complex Ru-**c** (26.2 mg, 0.015 mmol), NaOAc (8.2 mg, 0.10 mmol) and magnetic stirring bar were placed in a glass tube. The glass tube was inserted into an autoclave, which was closed tightly, evaporated in vacuum, and refilled with Ar gas. To the mixture was added anhydrous toluene (3 mL) under a continuous flow of Ar, and inside the autoclave was purged several times with H_2 gas (P_{H2} = 1.5 MPa). The autoclave was pressurized by H_2 gas (P_{H2} = 4 MPa) at room temperature, and heated at 160 ºC for 24 h with stirring (1000 rpm). The autoclave was cooled to 0 ºC in an ice–water bath. The reaction mixture was transferred into a 100 mL round bottom flask with $CHCl_3$ and concentrated under a reduced pressure (ca. 35 mmHg, 40 °C). The residue was dissolved in $CDCl_3$, and analyzed by ^{1}H NMR. The yields of 3-phenyl-1-propanol (AL-**a**) (90 %) and 1-(3-phenylpropyl) 3-phenylpropanoate (ES-**a**) (5 %) were calculated based on the integral ratio among the signals of these compounds with respected to an internal standard (mesitylene).

The ^{1}H NMR spectra of alcohols obtained in the hydrogenation experiments were consistent with the reported values: 3-phenyl-1-propanol (AL-**a**),[9] octadecanol (AL-**b**),[10] 3-cyclohexyl-1-propanol (AL-**c**),[11] 1-adamantanemethanol (AL-**d**),[12] 2-phenoxyethanol (AL-**e**),[13] *N*-(5-hydroxypentyl)benzamide (AL-**f**),[14] 2-(4-hydroxybutyl)thiophene (AL-**g**),[15] methyl 8-hydroxyoctanoate (AL-**h**),[16] methyl 4-(3-hydroxypropyl)benzoate (AL-**i**),[17] DL-2-Methyl-1-butanol (AL-**k**) (authentic sample), benzyl alcohol (AL-**l**),[18] (4-*tert*-butylphenyl)methanol (AL-**m**),[18] and [4-(trifluoromethyl)phenyl]methanol (AL-**n**).[18] Alcohols (AL-**b**, AL-**d**, AL-**h** and AL-**i**) were isolated by silica gel column chromatography (eluent: hexane/AcOEt = 10/1 (AL-**b**, 85 %), hexane/AcOEt = 8/1 (AL-**d**, 95 %), hexane/AcOEt = 3/1 (AL-**h**, 58 %), hexane/AcOEt = 2/1 (AL-**i**, 78 %)).

Representative hydrogenation procedure for investigation of effects of benzoic acid (CA-l) on reaction rate (CA-a = 0.60 mmol, CA-l = 0.20 mmol, Supplementary Figure 2, Entry 1)

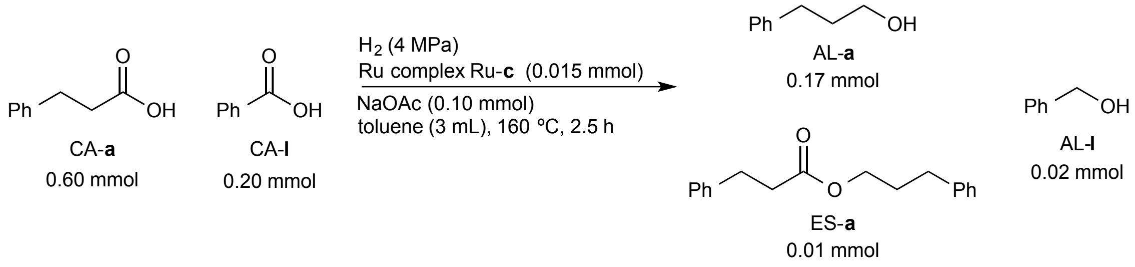

3-Phenylpropionic acid (CA-**a**) (90.1 mg, 0.60 mmol), benzoic acid (CA-**l**) (24.5 mg, 0.20 mmol), Ru complex Ru-**c** (26.2 mg, 0.015 mmol), NaOAc (8.2 mg, 0.10 mmol) and magnetic stirring bar were placed in a glass tube. The glass tube was inserted into an autoclave, which was closed tightly, evaporated in vacuum, and refilled with Ar gas. To the mixture was added anhydrous toluene (3 mL) under a continuous flow of Ar, and inside the autoclave was purged several times with H_2 gas (P_{H2} = 1.5 MPa). The autoclave was pressurized by H_2 gas (P_{H2} = 4 MPa) at room temperature, and heated at 160 ºC for 2.5 h with stirring (1000 rpm). The autoclave was cooled to 0 ºC in an ice–water bath. The reaction mixture was transferred into a 100 mL round bottom flask with $CHCl_3$ and concentrated under a reduced pressure (ca. 35 mmHg, 40 °C). The residue was dissolved in $CDCl_3$, and analyzed by ^{1}H NMR. The yield of 3-phenyl-1-propanol (AL-**a**) (0.17 mmol), 1-(3-phenylpropyl) 3-phenylpropionate (ES-**a**) (0.01 mmol) and benzylalcohol (AL-**l**) (0.02 mmol) were calculated based on the integral ratio among the signals of these compounds with respected to an internal standard (mesitylene).

Representative hydrogenation procedure for investigation of effects of substrate concentration (CA-l) on reaction rate (CA-l = 0.60 mmol, Supplementary Figure 3, Entry 4)

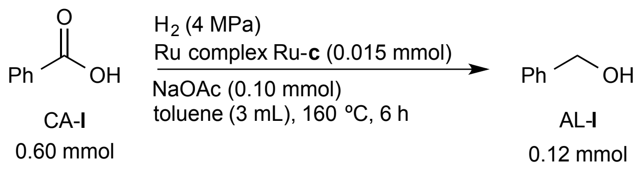

Benzoic acid (CA-**l**) (73.3 mg, 0.60 mmol), Ru complex Ru-**c** (26.2 mg, 0.015 mmol), NaOAc (8.2 mg, 0.10 mmol) and magnetic stirring bar were placed in a glass tube. The glass tube was inserted into an autoclave, which was closed tightly, evaporated in vacuum, and refilled with Ar gas. To the mixture was added anhydrous toluene (3 mL) under a continuous flow of Ar, and inside the autoclave was purged several times with H_2 gas (P_{H2} = 1.5 MPa). The autoclave was pressurized by H_2 gas (P_{H2} = 4 MPa) at room temperature, and heated at 160 ºC for 6 h with stirring (1000 rpm). The autoclave was cooled to 0 ºC in an ice–water bath. The reaction mixture was transferred into a 100 mL round bottom flask with $CHCl_3$ and concentrated under a reduced pressure (ca. 35 mmHg, 40 °C). The residue was dissolved in $CDCl_3$, and analyzed by ^{1}H NMR. The yield of benzylalcohol (AL-**l**) (0.12 mmol) was calculated based on the integral ratio among the signals of these compounds with respected to an internal standard (mesitylene).

Representative hydrogenation procedure for investigating the P_{H2} dependency using Ru complex Ru-e (P_{H2} = 1 MPa, Supplementary Figure 9, Entry 1)

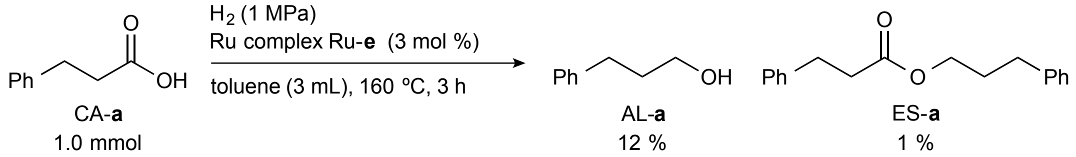

3-Phenylpropionic acid (CA-**a**) (150.2 mg, 1.0 mmol), Ru complex Ru-**e** (27.4 mg, 0.030 mmol) and magnetic stirring bar were placed in a glass tube. The glass tube was inserted into an autoclave, which was closed tightly, evaporated in vacuum, and refilled with Ar gas. To the mixture was added anhydrous toluene (3 mL) under a continuous flow of Ar, and inside the autoclave was purged several times with H_2 gas (P_{H2} = 1.5 MPa). The autoclave was pressurized by H_2 gas (P_{H2} = 1 MPa) at 25 ºC, and heated at 160 ºC for 3 h with stirring (1000 rpm). The autoclave was cooled to 0 ºC in an ice–water bath. The reaction mixture was transferred into a 100 mL round bottom flask with $CHCl_3$ and concentrated under a reduced pressure (ca. 35 mmHg, 40 °C). The residue was diluted with $CDCl_3$, and analyzed by ^{1}H NMR. The yields of 3-phenyl-1-propanol (AL-**a**) (12 %) and 1-(3-phenylpropyl) 3-phenylpropionate (ES-**a**) (1 %) were calculated based on the integral ratio among the signals of these compounds with respected to an internal standard (mesitylene).

Representative hydrogenation procedure in the screening of Ru complexes: Ru complex = $RuCl_2(PPh_3)_3$ (Ru-a) (Supplementary Table 1, Entry 1)

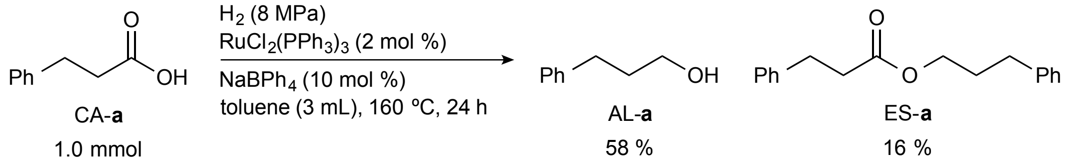

3-Phenylpropionic acid (CA-**a**) (150.2 mg, 1.0 mmol), $RuCl_2(PPh_3)_3$ (Ru-**a**) (19.2 mg, 0.020 mmol), $NaBPh_4$ (34.2 mg, 0.10 mmol) and magnetic stirring bar were placed in a glass tube. The glass tube was inserted into an autoclave, which was closed tightly, evaporated in vacuum, and refilled with Ar gas. To the mixture was added anhydrous toluene (3 mL) under a continuous flow of Ar, and inside the autoclave was purged several times with H_2 gas (P_{H2} = 1.5 MPa). The autoclave was pressurized by H_2 gas (P_{H2} = 8 MPa) at room temperature, and heated at 160 ºC for 24 h with stirring (1000 rpm). The autoclave was cooled to 0 ºC in an ice–water bath. The reaction mixture was transferred into a 100 mL round bottom flask with $CHCl_3$ and concentrated under a reduced pressure (ca. 35 mmHg, 40 °C). The residue was dissolved in $CDCl_3$, and analyzed by ^1H NMR. The yields of 3-phenyl-1-propanol (AL-**a**) (58 %) and 1-(3-phenylpropyl) 3-phenylpropionate (ES-**a**) (16 %) were calculated based on the integral ratio among the signals of these compounds with respected to an internal standard (mesitylene).

Representative hydrogenation procedure for the screening of phosphine ligands using *cis*-$RuCl_2(DMSO)_4$ and $NaBPh_4$ (Phosphine: PPh_3 (L-a), Supplementary Table 2, Entry 1)

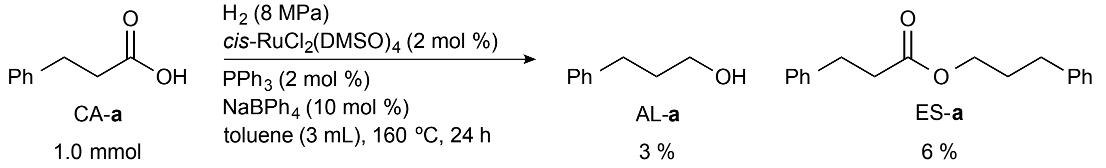

3-Phenylpropionic acid (CA-**a**) (150.2 mg, 1.0 mmol), *cis*-$RuCl_2(DMSO)_4$ (9.70 mg, 0.020 mmol), $NaBPh_4$ (34.2 mg, 0.10 mmol), triphenylphosphine (L-**a**) (5.25 mg, 0.020 mmol) and magnetic stirring bar were placed in a glass tube. The glass tube was inserted into an autoclave, which was closed tightly, evaporated in vacuum, and refilled with Ar gas. To the mixture was added anhydrous toluene (3 mL) under a continuous flow of Ar, and inside the autoclave was purged several times with H_2 gas (P_{H2} = 1.5 MPa). The autoclave was pressurized by H_2 gas (P_{H2} = 8 MPa) at room temperature, and heated at 160 ºC for 24 h with stirring (1000 rpm). The autoclave was cooled to 0 ºC in an ice–water bath. The reaction mixture was transferred into a 100 mL round bottom flask with $CHCl_3$ and concentrated under a reduced pressure (ca. 35 mmHg, 40 °C). The residue was dissolved in $CDCl_3$, and analyzed by ^{1}H NMR. The yields of 3-phenyl-1-propanol (AL-**a**) (3 %) and 1-(3-phenylpropyl) 3-phenylpropionate (ES-**a**) (6 %) were calculated based on the integral ratio among the signals of these compounds with respected to an internal standard (mesitylene).

Representative hydrogenation procedure for optimization of reaction conditions using Ru complex Ru-c (additive: NaOAc; solvent: toluene, Supplementary Table 3, Entry 2)

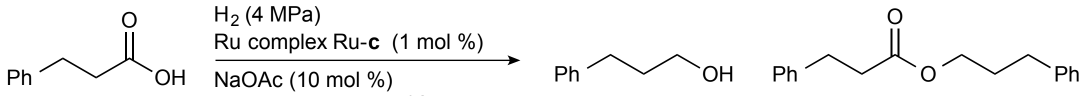

CA-**a** 1.0 mmol; AL-**a** 62 %; ES-**a** 15 %

3-Phenylpropionic acid (CA-**a**) (150.2 mg, 1.0 mmol), Ru complex Ru-**c** (17.5 mg, 0.010 mmol), NaOAc (8.2 mg, 0.10 mmol) and magnetic stirring bar were placed in a glass tube. The glass tube was inserted into an autoclave, which was closed tightly, evaporated in vacuum, and refilled with Ar gas. To the mixture was added anhydrous toluene (3 mL) under a continuous flow of Ar, and inside the autoclave was purged several times with H_2 gas (P_{H2} = 1.5 MPa). The autoclave was pressurized by H_2 gas (P_{H2} = 4 MPa) at room temeprature, and heated at 160 ºC for 24 h with stirring (1000 rpm). The autoclave was cooled to 0 ºC in an ice–water bath. The reaction mixture was transferred into a 100 mL round bottom flask with $CHCl_3$ and concentrated under a reduced pressure (ca. 35 mmHg, 40 °C). The residue was dissolved in $CDCl_3$, and analyzed by ^{1}H NMR. The yields of 3-phenyl-1-propanol (AL-**a**) (62 %) and 1-(3-phenylpropyl) 3-phenylpropionate (ES-**a**) (15 %) were calculated based on the integral ratio among the signals of these compounds with respected to an internal standard (mesitylene).

Representative hydrogenation procedure for investigating the effects of water using Ru complex Ru-c/NaBPh$_4$/H$_2$O (0.5 mL) (Supplementary Table 4, Entry 2)

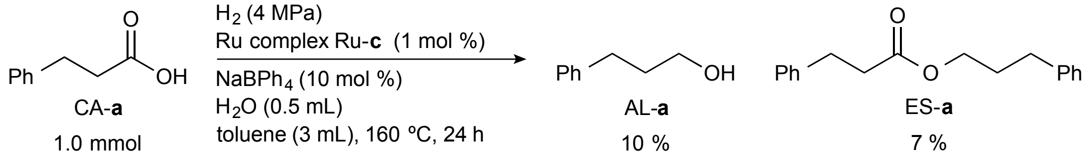

3-Phenylpropionic acid (CA-**a**) (150.2 mg, 1.0 mmol), Ru complex Ru-**c** (17.5 mg, 0.010 mmol), NaBPh$_4$ (8.2 mg, 0.10 mmol) and magnetic stirring bar were placed in a glass tube. The glass tube was inserted into an autoclave, which was closed tightly, evaporated in vacuum, and refilled with Ar gas. To the mixture was added anhydrous toluene (3 mL) and H_2O (0.5 mL) under a continuous flow of Ar, and inside the autoclave was purged several times with H_2 gas (P_{H2} = 1.5 MPa). The autoclave was pressurized by H_2 gas (P_{H2} = 4 MPa) at room temperature, and heated at 160 ºC for 24 h with stirring (1000 rpm). The autoclave was cooled to 0 ºC in an ice–water bath. The reaction mixture was transferred into a 100 mL round bottom flask with $CHCl_3$ and concentrated under a reduced pressure (ca. 35 mmHg, 40 °C). The residue was dissolved in $CDCl_3$, and analyzed by ^{1}H NMR. The yields of 3-phenyl-1-propanol (AL-**a**) (10 %) and 1-(3-phenylpropyl) 3-phenylpropionate (ES-**a**) (7 %) were calculated based on the integral ratio among the signals of these compounds with respected to an internal standard (mesitylene).

5. Control experiments

Investigation of a possibility of esterification

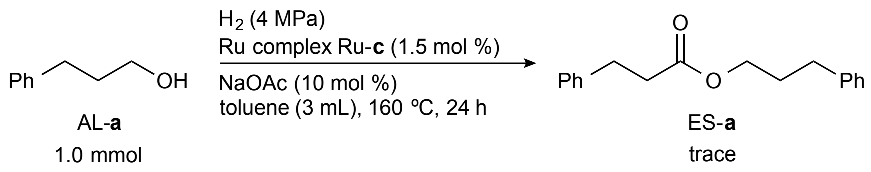

Ru complex Ru-**c** (26.2 mg, 0.015 mmol), NaOAc (8.2 mg, 0.10 mmol) and magnetic stirring bar were placed in a glass tube. The glass tube was inserted into an autoclave, which was closed tightly, evaporated in vacuum, and refilled with Ar gas. To the mixture was added 3-phenyl-1-propanol (AL-**a**) (136 μL, 1.0 mmol) and anhydrous toluene (3 mL) under a continuous flow of Ar, and inside the autoclave was purged several times with H_2 gas (P_{H2} = 1.5 MPa). The autoclave was pressurized by H_2 gas (P_{H2} = 4 MPa) at room temperature, and heated at 160 ºC for 24 h with stirring (1000 rpm). The autoclave was cooled to 0 ºC in an ice–water bath. The reaction mixture was transferred into a 100 mL round bottom flask with $CHCl_3$ and concentrated under a reduced pressure (ca. 35 mmHg, 40 °C). The residue was diluted with $CDCl_3$, and analyzed by ^{1}H NMR. The yield of 1-(3-phenylpropyl) 3-phenylpropionate (ES-**a**) (trace) was calculated based on the integral ratio among the signals of these compounds with respected to an internal standard (mesitylene).

Investigation of active species generated from Ru complex Ru-c and NaOAc (Supplementary Figure 4)

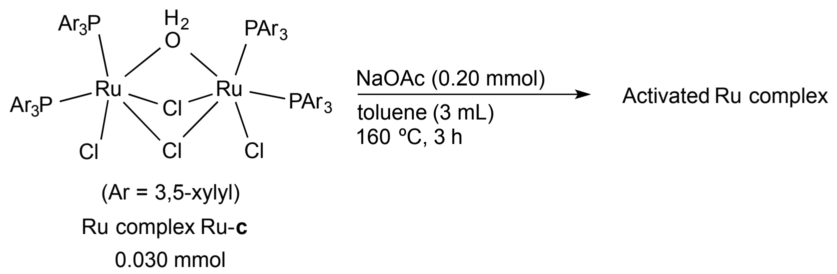

Ru complex Ru-**c** (52.4 mg, 0.030 mmol), NaOAc (16.4 mg, 0.20 mmol) and magnetic stirring bar were placed in a glass tube. The glass tube was inserted into an autoclave, which was closed tightly, evaporated in vacuum, and refilled with Ar gas. To the mixture was added anhydrous toluene (3 mL) under a continuous flow of Ar and heated at 160 ºC for 3 h with stirring (1000 rpm). The autoclave was cooled to 0 ºC in an ice–water bath. The reaction mixture was diluted with acetonitrile and analyzed by ESI-MS.

Representative procedure for investigating the chemical species generated from Ru complex Ru-c, additive and 3-phenylpropionic acid (CA-a) (Additive: NaOAc, Supplementary Figure 5)

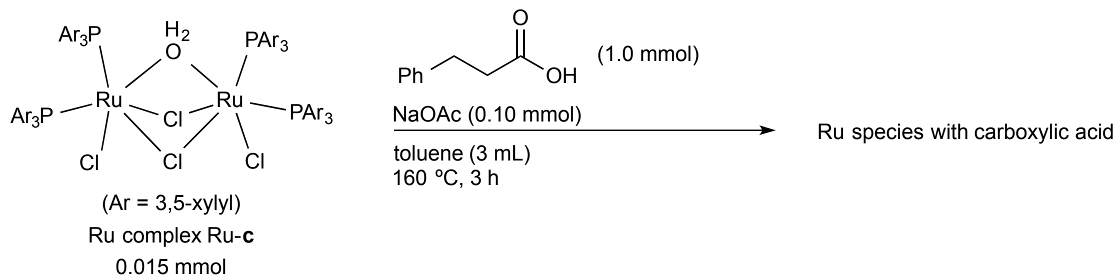

Ru complex Ru-**c** (26.2 mg, 0.015 mmol), NaOAc (8.2 mg, 0.10 mmol), 3-phenylpropinic acid (CA-**a**) (150.2 mg, 1.0 mmol) and magnetic stirring bar were placed in a glass tube. The glass tube was inserted into an autoclave, which was closed tightly, evaporated in vacuum, and refilled with Ar gas. To the mixture was added anhydrous toluene (3 mL) under a continuous flow of Ar and heated at 160 ºC for 3 h with stirring (1000 rpm). The autoclave was cooled to 0 ºC in an ice–water bath. The reaction mixture was diluted with acetonitrile and analyzed by ESI-MS.

Representative procedure for investigating the chemical species generated from Ru complex Ru-d, H_2, additive and 3-phenylpropionic acid (CA-a) (Additive: Na(acac)•xH_2O, Supplementary Figure 7)

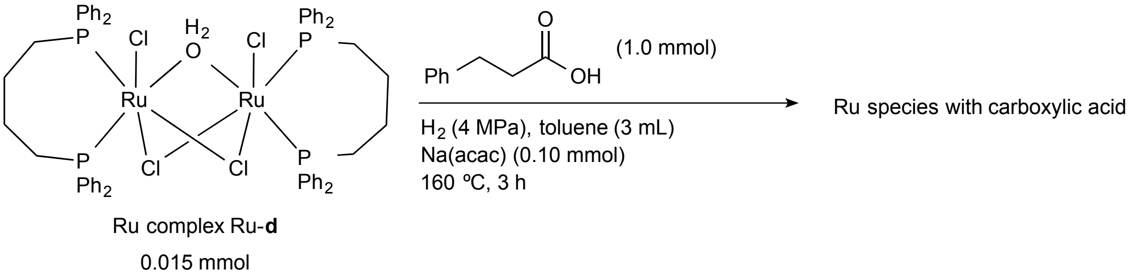

Ru complex Ru-**d** (18.2 mg, 0.015 mmol), Na(acac)•xH_2O (12.2 mg, 0.10 mmol), 3-phenylpropinic acid (CA-**a**) (150.2 mg, 1.0 mmol) and magnetic stirring bar were placed in a glass tube. The glass tube was inserted into an autoclave, which was closed tightly, evaporated in vacuum, and refilled with Ar gas. To the mixture was added anhydrous toluene (3 mL) under a continuous flow of Ar, and inside the autoclave was purged several times with H_2 gas (P_{H2} = 1.5 MPa). The autoclave was pressurized by H_2 gas (P_{H2} = 4 MPa) at room temperature, and heated at 160 ºC for 3 h with stirring (1000 rpm). The autoclave was cooled to room temperature in a water bath. The reaction mixture was diluted with acetonitrile and analyzed by ESI-MS.

Investigation of chemical species generated from Ru complex Ru-f, H_2 and 3-phenylpropionic acid (CA-a) (Supplementary Figure 8)

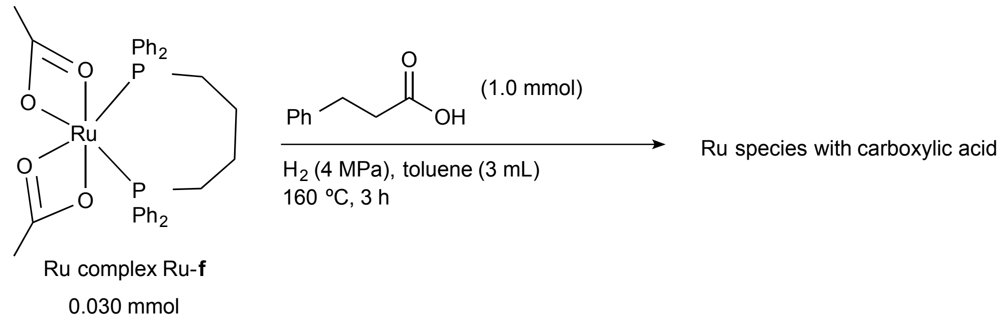

Ru complex Ru-**f** (19.4 mg, 0.030 mmol), 3-phenylpropinic acid (CA-**a**) (150.2 mg, 1.0 mmol) and magnetic stirring bar were placed in a glass tube. The glass tube was inserted into an autoclave, which was closed tightly, evaporated in vacuum, and refilled with Ar gas. To the mixture was added anhydrous toluene (3 mL) under a continuous flow of Ar, and inside the autoclave was purged several times with H_2 gas (P_{H2} = 1.5 MPa). The autoclave was pressurized by H_2 gas (P_{H2} = 4 MPa) at room tempperature, and heated at 160 ºC for 3 h with stirring (1000 rpm). The autoclave was cooled to room temperature in a water bath. The reaction mixture was diluted with acetonitrile and analyzed by ESI-MS.

X-ray single crystal structure analysis of Ru-**c (Supplementary Figure 1)**

Ru complex Ru-**c** was obtained as a single crystal, which was suitable for the X-ray single crystal analysis.

Intensity data were collected at 123 K on a Rigaku Single Crystal CCD X-ray Diffractometer (Saturn 70 with MicroMax-007) with Mo Kα radiation (λ = 0.71075 Å) and graphite monochromater. A total of 29170 reflections were measured at a maximum 2θ angle of 50.0°, of which 7937 were independent reflections (R_{int} = 0.0498). The structure was solved by direct methods (SHELXS-97) and refined by the full-matrix least-squares on F_2 (SHELXL-97). All non-hydrogen atoms were refined anisotropically. All hydrogen atoms were placed using AFIX instructions. The following crystal structure has been deposited at the Cambridge Crystallographic Data Centre and allocated the deposition number CCDC 1024070.

The crystal data are as follows: $C_{98}H_{116}Cl_4O_2P_4Ru_2$; FW = 1793.72, crystal size 0.20 × 0.20 × 0.20 mm^3, monoclinic, C2/c, a = 22.141(5) Å, b = 14.879(3) Å, c = 27.748(6) Å, α = 90 °, β = 95.582 (3)°, γ = 90 °, V = 9109 (3) Å^3, Z = 4, Dc = 1.308 g cm^{-3}. The refinement converged to R_1 = 0.0440, wR_2 = 0.0901 ($I > 2\sigma(I)$), GOF = 1.095.

Supplementary References

1. Reger, D. L., Wright, T. D., Little, C. A., Lamba, J. J. S. & Smith, M. D. Control of the stereochemical impact of the lone pair in lead(II) tris(pyrazolyl)methane complexes. Improved preparation of Na{B[3,5-$(CF_3)_2C_6H_3]_4$}. *Inorg. Chem.* **40,** 3810–3814 (2001).
2. Yakelis, N. A. & Bergman, R. G. Safe preparation and purification of sodium tetrakis[(3,5-trifluoromethyl)phenyl]borate ($NaBArF_{24}$): Reliable and sensitive analysis of water in solutions of fluorinated tetraarylborates. *Organometallics*, **24,** 3579–3581 (2005).
3. Jung, C. W., Garrow, P. E., Hoffman, P. R. & Caulton, K. G. Reexamination of the reactions of $Ph_2P(CH_2)_nPPh_2$ (n= 1-4) with $RuCl_2(PPh_3)_3$. *Inorg. Chem.* **23,** 726–729 (1984).
4. Joshi, A. M., Thorburn, I. S., Rettig, S. J. & James, B. R. Synthesis, characterization and reactivity of some mono- and dinuclear chlororuthenium complexes containing chelating ditertiary phosphines (P–P) with P–P:Ru = 1. *Inorg. Chim. Acta* **198,** 283–296 (1992).
5. Pechlivanidis, Z., Hopf, H. & Ernst, L. Paracyclophanes: extending the bridges. Synthesis. *Eur. J. Org. Chem.* 223–237 (2009).
6. Wong, W.-K., Lai, K.-K., Tse, M.-S., Tse. M.-C., Gao, J.-X., Wong, W.-T. & Chan, S. Reactivity of $Ru(OAc)_2(Ph_3P)_2$ toward chelating diphosphine ligands. X-ray crystal structures of *fac*-$Ru(OAc)_2(Ph_3P)$(dppm) and *trans*-$Ru(OAc)_2(P_2N_2H_4)$ *Polyhedron* **13,** 2751–2762 (1994).
7. Lynam, J. M., Welby, C. E. & Whitwood, A. C. Exploitation of a chemically non-innocent acetate ligand in the synthesis and reactivity of ruthenium vinylidene complexes. *Organometallics* **28,** 1320–1328 (2009).
8. MacFarlane, K. S., Thorburn, I. S., Cyr, P. W., Chau, D. E. K.-Y., Rettig, S. J. & James, B. R. Triply-bridged diruthenium(II) 1,4-bis(diphenylphosphino)butane (dppb) and (*o*)-2,2′-bis(diphenylphosphino)-1,1′-binaphthyl (binap) complexes, including structural characterisation of [(dppb) ClRu(μ-D_2O)-(μ-Cl)$_2$RuCl(dppb)], [(η^2-H_2) (dppb)Ru(μ-Cl)$_3$RuCl(dppb)] and the [(dppb)ClRu(μ-Cl)$_3$RuCl(dppb)]$^-$ anion. *Inorg. Chim. Acta* **270,** 130–144 (1998).

9. Murphy, J. A., Schoenebeck, F., Findlay, N. J., Thomson, D. W., Zhou, S.-Z. & Garnier. J. One-carbon extrusion from a tetraazafulvalene. Isolation of aldehydes and a study of their origin. *J. Am. Chem. Soc.* **131,** 6475–6479 (2009).
10. Iwasaki, T., Agura, K., Maegawa, Y., Hayashi, Y., Ohshima, T. & Mashima, K. A tetranuclear-zinc-cluster-catalyzed practical and versatile deprotection of acetates and benzoates. *Chem. Eur. J.* **16,** 11567 – 11571 (2010).
11. Kelly, C. B., Mercadante, M. A., Wiles, R. J. & Leadbeater, N. E. Oxidative esterification of aldehydes using a recyclable oxoammonium salt. *Org. lett.* **15,** 2222–2225 (2013).
12. Bodnar, B. S. & Vogt, P. F. An improved Bouveault−Blanc ester reduction with stabilized alkali metals. *J. Org. Chem.* **74,** 2598–2600 (2009).
13. David, S. & Thieffry, A. Conversion of one hydroxy group in a diol to a phenyl ether with triphenylbismuth diacetate, a new glycol reaction showing strong axial preference in six-membered rings. *J. Org. Chem.* **48,** 441–447 (1983).
14. Ghosh, S. C., Ngiam, J. S. Y., Seayad, A. M., Tuan, D. T., Chai, C. L. L. & Chen, A. Copper-catalyzed oxidative amidation of aldehydes with amine salts: synthesis of primary, secondary, and tertiary amides. *J. Org. Chem.* **77,** 8007–8015 (2012).
15. Afzali-Ardakani, A. Soluble sextiophene derivative and thin-film field effect transistor using the same. JP 2002100789 A (2002).
16. Yamamoto, N., Obora, Y. & Ishii, Y. Iridium-catalyzed oxidative methyl esterification of primary alcohols and diols with methanol. *J. Org. Chem.* **76,** 2937–2941 (2011).
17. Taylor, E. C. & Gillespie, P. Further acyclic analogs of 5,10-dideaza-5,6,7,8-tetrahydrofolic acid. *J. Org. Chem.* **57,** 5757–5761 (1992).
18. Dieskau, A. P., Begouin, J.-M. & Plietker, B. $Bu_4N[Fe(CO)_3(NO)]$-catalyzed hydrosilylation of aldehydes and ketones. *Eur. J. Org. Chem.* 5291–5296 (2011).
